# Building a physics-based virtual refrigerated container filled with fruit in ventilated packaging

**DOI:** 10.1016/j.mex.2024.102984

**Published:** 2024-09-27

**Authors:** Thijs Defraeye, Celine Verreydt, Julien Gonthier, Leo Lukasse, Paul Cronjé, Tarl Berry

**Affiliations:** a Swiss Federal Laboratories for Materials Science and Technology, Laboratory for Biomimetic Membranes and Textiles, Empa, Lerchenfeldstrasse 5, CH-9014 St., Gallen, Switzerland; b Food Quality and Design, Wageningen University & Research, P.O. Box 17, Wageningen 6700 AA, the Netherlands; c Wageningen University and Research, Bornse Weilanden 9, Wageningen 6708 WG, the Netherlands; d Citrus Research International, Nelspruit, South Africa; e Department of Horticultural Science, Stellenbosch University, Stellenbosch, South Africa

**Keywords:** Multiphysics, Computational, Citrus, Digital twin, Refrigeration, Slot-ventilated enclosure, Method to build a physics based model of a refrigerated container

## Abstract

We build a validated physics-based model of a refrigerated container filled with fruit in ventilated packaging. This model of a virtual container is the basis for simulations in an accompanying paper on citrus fruit shipped overseas from South Africa to Europe. The model is used to understand better how the cargo cools and when and where food quality is lost in these supply chains. We build a computational fluid dynamics model with a two-phase porous media approach that simulates the airflow in the container and the cooling process of every fruit. This container can be considered aerodynamically to be a slot-ventilated enclosure. We also model the fruit's thermally-driven quality loss. Using a two-phase porous media approach for the ventilated cargo and modeling temperature-driven fruit quality evolution are two steps forward compared to most existing physics-based refrigerated container models. We validate the porous media model implementation. We define and apply actionable metrics for every fruit inside the cargo, such as remaining shelf life upon arrival and seven-eighths cooling time.

• This model can help reduce food loss and increase supply-chain resilience.
• This model is an essential building block of a refrigerated container’s digital twin.
• This model can support stakeholders in improving cargo temperature control and resulting fruit quality preservation.

## Graphical abstract

**Figure:**
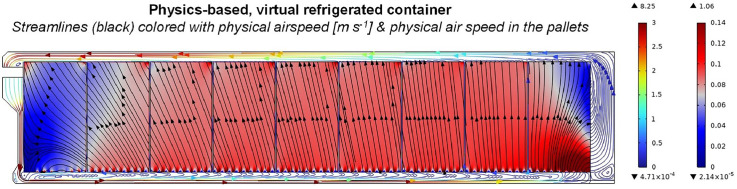

Specifications table

**Table:** 

Subject area:	
More specific subject area:	Refrigerated storage of fresh produce
Name of your method:	Method to build a physics based model of a refrigerated container
Name and reference of original method:	Not applicable
Resource availability:	Not applicable

## Method details

### Materials and methods

#### Container, packaging, palletization, and cold chain specifications

##### Packaging

###### Box

**Fig. 1:**
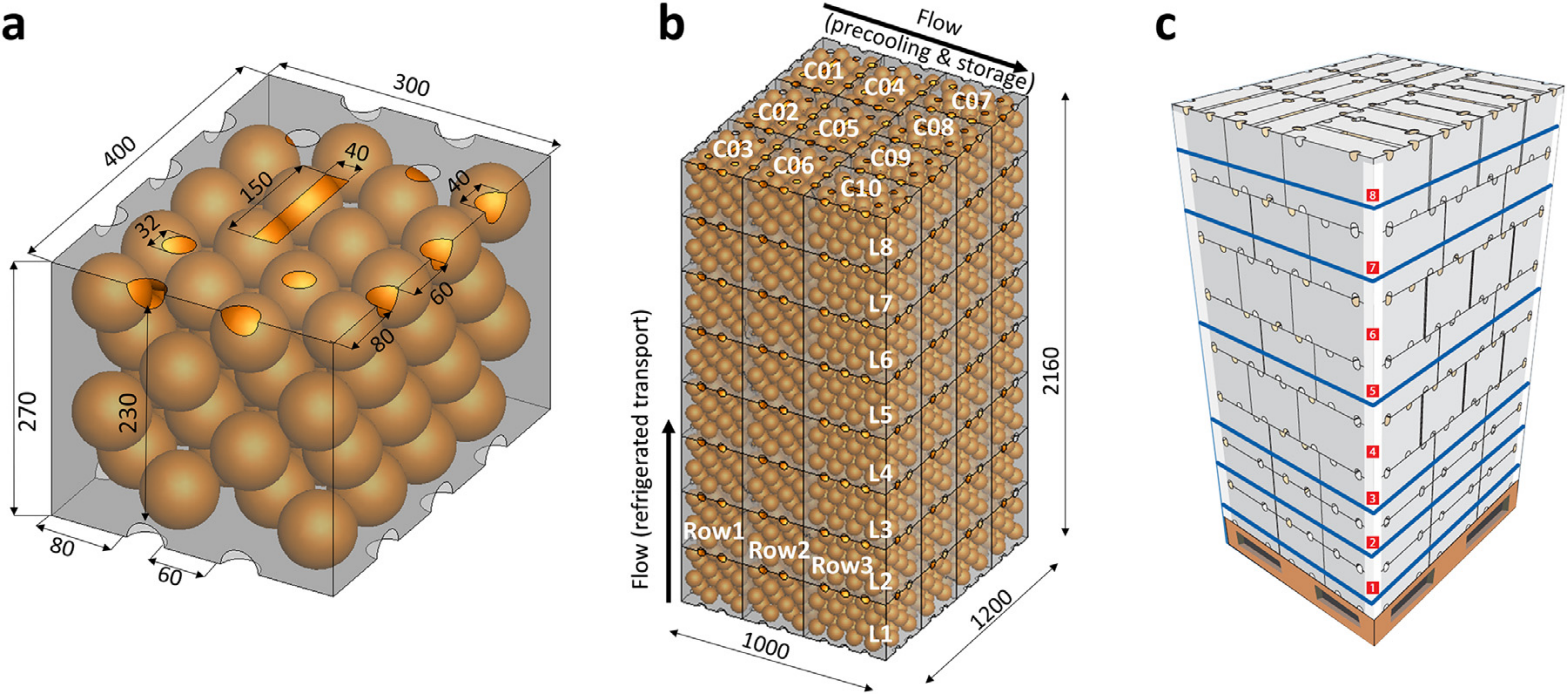
(a) Geometry and dimensions of the Supervent carton used in the present study and by ([1,2]a). (b) Illustration of idealized stacking of the cartons for an 8-layer, high-cube citrus pallet (10 Supervent cartons per layer, each packed with 16.5 kg of fruit), together with typical airflow for forced-air precooling (horizontal flow) and in a refrigerated container (vertical airflow). (c) Illustration of actual stacking used in commercial operations with a pallet base. Note that a slightly different design of the Supervent carton is shown here, compared to the one shown in (a), with fewer ventilation holes. Dimensions are in mm.

A telescopic corrugated fiberboard carton type ‘A15C Supervent’ was used (0.4 × 0.3 × 0.27 m, Fig. 1a). The carton contains ‘Navel’ oranges (*Citrus senensis* L. Osb). The citrus fruit is spherical and has a diameter of 80 mm. The carton box has half-circular vent holes located at the top and bottom of each side. Note that newer designs of this carton type have been developed in the past years. This carton was already evaluated for forced airflow cooling [1,2]. The carton's total open area (TOA) on the long vertical side, short vertical side, bottom, and top is 3.5 %, 3.1 %, 9.1 %, and 12.9 %, respectively. All fruit are placed in the carton according to a staggered pattern. The Supervent cartons hold 64 orange fruit and have a mass of 16.5 kg each. The amount of fruit in the box is 53 % of the total box volume. The corresponding porosity is 47 % (ε_fr_). This porosity includes the volume of air and the packaging material as 'air space' and only the fruit as the solid material. The porosity, when including the package and the fruit as a solid material (ε), is lower and equals 38 %.

###### Carton

The Supervent cartons are stacked to assemble a high-cube pallet (1.2 m x 1.0 m x 2.16 m, Fig. 1b). On a pallet, 8 layers of cartons are stacked, where each layer holds 10 cartons (Fig. 1b). In total, 3 rows are present (Row1, Row2, and Row3) along the horizontal flow path for precooling and cold storage, and 8 layers (L1-L8) are present along the vertical flow path for refrigerated container transport. Each layer contains 10 cartons (C01-C10). Row1 and Row2 have 3 cartons for each layer, whereas Row3 has 4 cartons (Fig. 1b). The pallet contains 80 cartons and holds 5120 fruit. The ‘Supervent’ package was designed to form vertical and horizontal ventilation pathways via the vent holes during precooling and transport after palletization. The airflow direction is undefined when the produce is transferred between the two unit operations. The air temperature that enters the pallet will depend on the environment in which the pallet is placed, and it should preferably be cooled. Though this carton design also enables vertical pathways, these are less evident in practice due to the staggered stacking pattern of consecutive layers (Fig. 1c). Practitioners deem this necessary to ensure the stability of the pallet during handling and transport, but it obstructs several vent holes. In addition, the slats of the wooden pallet base fully or partially cover some of the vent holes of the bottom carton box (Fig. 1c).

###### Pallet base

**Fig. 2:**
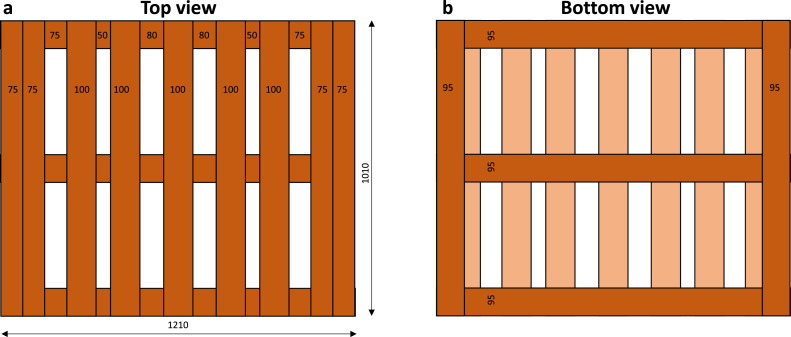
Pallet base top view and bottom view with dimensions (in mm).

The wooden pallets (Fig. 2) on which the cartons are stacked measure 1.21 m x 1.01 m x 0.16 m. This pallet is common in the South African citrus industry. The bottom part of the pallet base has an open area of 60 % of the total surface area of the pallet (1.01 × 1.21 m). The top part of the pallet base, which is in contact with the fruit cartons, has an open area of only 24 % of the pallet base. Other pallets can have an even smaller open area (15 % for [3]). The wooden blocks between the top and bottom of the pallet base have an open area (or also open volume) of about 90 % of the pallet area. The pallet base provides a significant blockage to the airflow, especially the top part of the pallet base. Compared to the pallet of fruit in ventilated packaging, the resulting pressure loss over the pallet base is, however, rather small.

#### Container

##### Dimensions

**Fig. 3:**
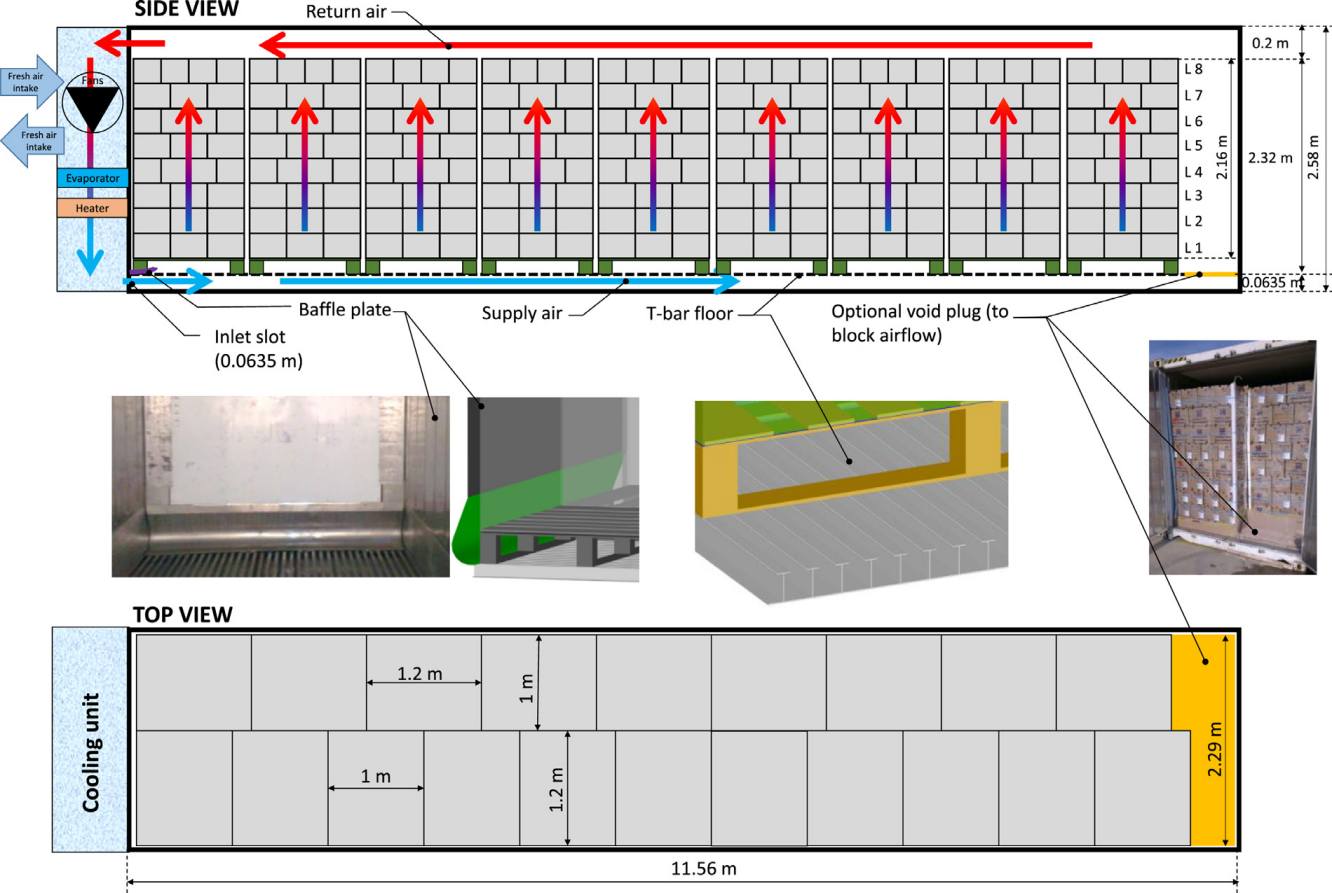
Side and top cross-sectional views of a high-cube refrigerated container with an indication of the stacking pattern of the pallets and the box layers. The inner dimensions of the container are shown.

A state-of-the-art reefer container was used in the present study (Fig. 3). Such refrigerated containers have a lifetime of about 12–18 years [4]. The container details are specified in [5]. This 40-foot HC (High-Cube) container had inner dimensions of 11.56 × 2.29 × 2.58 m and external dimensions of 12.19 × 2.43 × 2.90 m. These inner dimensions do not include the T-bar floor, which is installed afterward. This floor is composed of extruded T-bars that go from the side of the refrigeration unit to the container door side (35 full T-bars of 63.5 mm height and 63.5 mm width). The container was filled with 20 pallets of fruit (1 × 1.2 m). The loading pattern is shown in Fig. 3, but other loading patterns are also possible. In this study, the container contains 1600 cartons and 102,400 fruit. Due to an inherent mismatch between the dimensions of the pallets with those of the container, not the entire floor area can be used. Even when filled with standard pallets (pallet base = 1.01 × 1.21 m^2^), 7.7 % of the container's floor area remains uncovered (2.03 m^2^). This area is located mostly at the door end of the container. However, on the lateral sides of the container, a gap of 70 mm is present for such pallets. This is usually required to provide some clearance when loading the pallets, as they are not perfectly stacked to a 1.01 × 1.21 m size.

Nevertheless, the open area on the lateral sides remains. Also, note that the pallets of fruit, so the packages on the pallet base, only occupy 1.0 × 1.2 m when stacked ideally. The space on the lateral sides of the container for the packages is 90 mm since the container is 2290 mm wide and two pallets only occupy 2200 mm. In practice, pallets tend to bulge and thus become a bit wider after handling. As such, the actual gap size will likely be less than 90 mm and irregularly distributed. The gaps will mostly be on the center line between the two pallet rows because it is more convenient for the forklift driver during loading. The air enters the container via the bottom through an inlet slot of a height of 63.5 mm [5]. This inlet slot has the same height as the T-bar floor since the air is directed from the refrigeration unit via a baffle plate into the T-bar floor.

##### Airflow generation & airflow rate

Airflow in the container is generated by the two axial evaporator fans in the refrigeration unit [6]. These fans circulate air through the container along the bottom via a grated T-bar floor. Air is drawn vertically upwards through the cargo and returns to the refrigeration unit via the ceiling (Fig. 3). Note that the airflow direction is mostly vertical, but horizontal airflow components can also be present, which are dependent on the location and the distance from the inlet. For fresh fruit and vegetables, the air is forced through the cargo, packed in ventilated packaging, to dissipate possible respiratory heat and metabolic gasses (e.g., carbon dioxide and ethylene). For frozen cargo, however, the air is directed around the cargo. Typically two fans are installed. These fans operate continuously but are briefly switched off during a defrosting cycle. Nevertheless, the container's evaporator fans typically have two operating regimes for which a different RPM can be set. For the Carrier Transicold container in this study, the two evaporator axial fans can run at two speeds: high (max) speed, so an RPM of 3460/2850 driven at 62/50 Hz, and low (half) speed, so an RPM of 1760/1425 driven at 62/50 Hz motor, and no speed, when the fans are switched off. These fans are driven by a 3-phase (380/460 V) 62/50 Hz motor. There are two operating modes of these fans for a container: chilled mode (> ∼ -5°C) and frozen mode (< ∼ -5°C [7]). Typically, in chilled mode, the evaporator fans run continuously at high speed, and the compressor operates continuously. In frozen mode, the evaporator fans run at a low speed, and the refrigeration system works in on/off control [4].

Both container fans typically deliver a total flow rate of 1800–5500 m^3^ h^−1^ together, depending on whether the container is loaded and on the selected speed regime of the evaporator fans. The corresponding air exchange rate lies between about 25–80 air changes per hour of the internal volume of the container at low and high speeds, respectively. This amount of air exchange is large, but note that not all regions of the container are as well ventilated. Therefore, care should always be taken to ensure proper ventilation in all locations and avoid developing hot spots. The airflow conditions in a loaded container that were used in this study are detailed in Table 1 and were measured in a loaded container at different operating regimes at 50 Hz and 62 Hz [8]. These conditions result in an average airspeed of 3.4–7.9 m s^−1^ at the inlet slot (0.0635 × 2.29 m^2^) and 0.019–0.044 m s^−1^ over the container floor area (Table 1).

**Table 1:** Specifications of airflow in a loaded high-cube refrigerated container (for 2 evaporator fans).

Airflow regime	Airflow rate [m^3^ h^−1^]	Fan pressure rise [Pa]	Fan power [W]	Average airspeed over the container floor area x 10^3^ [m s^−1^]	Flow rate per kg of fruit [L s^−1^kg^−1^]	Airspeed at inlet slot [m s^−1^]
Low speed @ 62Hz (50Hz)	2175 (1770)	55 (37)	270 (200)	22.8 (18.6)	0.023 (0.019)	4.2 (3.4)
High speed @ 62Hz (50Hz)	4150 (3490)	198 (146)	1710 (940)	43.5 (36.6)	0.044 (0.037)	7.9 (6.7)

Nowadays, however, the low-speed regime is used to save energy and reduce fan heat production and is used in frozen or chilled mode with non/low-respiring fruit and vegetables. With more advanced cooling protocols, the controller often chooses the low-speed regime based on the measured temperature difference between supply and return air [9,10]. This reduces the energy consumption of the fans in chilled mode by optimizing evaporator fan speed based on the heat load and also avoids inefficient part-load compressor operation while still maintaining quality [9,10].

##### Evaporator fans

**Fig. 4:**
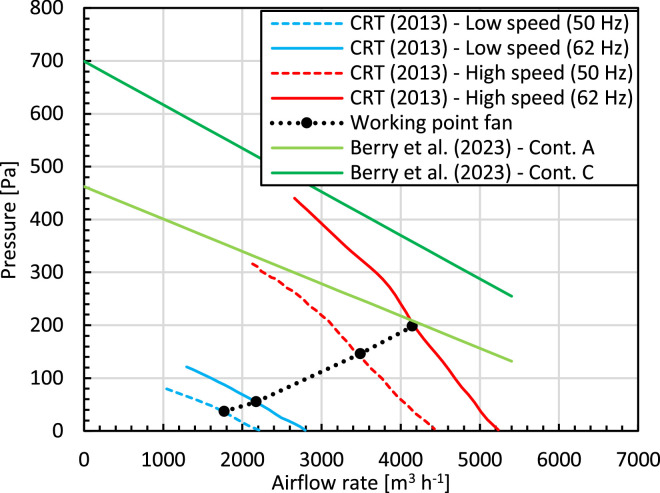
Fan performance curve (for 2 fans together), so airflow rate through these fans as a function of the fans' static pressure rise from two sources [8,11]. The system curve with individual working points of the fans for a loaded container is also shown [8].

Fans are typically characterized by their fan performance curve. This curve displays several airflow rates and the static pressure rise that the fan can deliver. This curve is determined by measuring the air flow rate through a partially obstructed channel where the fan is placed. By incrementally increasing the aerodynamic resistance of the channel, so increasing the obstruction, different airflow rates are obtained at different static pressures. Typical fan performance curves for the two fans installed in a refrigerated container are depicted in Fig. 4 [8,11,12]. Note that these fan performance curves are independent of the airflow resistance of the system in which the fan is placed.

##### Airflow resistance that the fan needs to overcome

The actual airflow rate and the pressure increase the fans provide are, to some extent, dependent on the pressure resistance of the cargo and the rest of the pressure losses in the closed circuit of the container. The aerodynamic (airflow) resistance of the container circuit is expressed as the relation between the total pressure drop over the container circuit from the fan outlet to the fan inlet (*∆P_t_* [Pa]) and the volumetric air flow rate through the fans (*G_a_* [m^3^ s^−1^]):

(1) $$\Delta P_{t}=\sum_{i}\Delta P_{t,i}=\xi_{tot}G_{a,fan}^{2}$$

Here *ξ* is the pressure loss coefficient of the entire circuit ([Pa s^2^ m^−6^] or [kg m^−7^]). This pressure drop only accounts for inertial effects (Forchheimer term). The viscous effects (Darcy term, proportional to *G_a_*) only become important at very low flow speeds [13] and are negligible in most parts of the container due to the high speeds. It should be noted that the total pressure (*P_t_*) is the sum of the static pressure (*P_s_*) and the dynamic pressure (*P_d_* = *0.5 ρ U^2^*, with *ρ* the air density [kg m^−3^] and *U* the airspeed [m s^−1^]). The airspeed upstream and downstream of the fan is the same, so *P_d_* remains constant by which *∆P_t_* = *∆P_s_* in Eq. (1) [14]. However, the dynamic pressure will change throughout the airflow circuit of the refrigerated container as speeds in the cargo hold are much lower.

All system components that cause pressure losses in the closed circuit of the container determine the container's system curve. The intersection of the system curve with the fan performance curve determines the corresponding working point of the system (*∆P_t,wp_*(*G_a,wp_*)). The system curve for a loaded container with individual working points at different speeds is shown in Fig. 4. The contribution of palletized fruit to the system curve, so to the working point *∆P_t,wp_*(*G_a,wp_*) and the total power consumed by the fan (*P_w,fan_*), can be substantial. This is often the case for forced airflow cooling [15], where the product-packaging ensemble is one of the main resistances in the system. For refrigerated containers, though, the pressure loss coefficient of the pallets is high, but the airspeed through them is much lower than in other components in the refrigeration unit. Thereby the contribution of the palletized fruit to the resulting pressure loss over the cargo hold is rather small (∼ 10^0^-10^1^ Pa, from our study and other work reported in [8]) compared to the pressure rise provided by the fan (∼ 50–200 Pa, Table 1, Fig. 4). The cargo, and thus the fruit geometry, packaging density, and packaging are relevant but not-dominant determinants in the aerodynamic resistance the fans need to overcome. The main aerodynamic resistance lies in the refrigeration unit. However, still, with an empty container, the fans can run at a different speed then for a filled container, due to the contribution of the cargo to the pressure loss over the entire circuit.

##### Fan power consumption

The total power consumed by the fan (*P_w,fan_*) can be calculated as: *P_w,fan_ = ∆P_t,wp_ G_a,wp_/(η_fan_ η_motor_*), where wp indicates the working point of the fan. *P_w,fan_* also depends on the fan and motor efficiencies (*η_fan_* and *η_motor_* [16]) and is thereby proportional to *G_a,wp_^3^*. The fan energy consumption (*E_fan_* [J]) can easily be calculated as *E_fan_* = *P_w,fan_ t_op_*, with *t_op_* the operational time of the fan.

The power required to push air through the ventilated cargo (*P_w,cargo_* [W]) can be determined [1,17]:

(2) $$P_{w,c\arg o}=\Delta P_{t,c\arg o}G_{a}=\xi_{c\arg o}G_{a}^{3}$$

*P_w_* only contains here the contribution of the packaging. Therefore the aerodynamic resistance of other system components has to be accounted for as well to determine the system curve (similar to Eq. (9) [14,18]). As mentioned, the pressure losses over the other components will likely dominate the pressure the fans need to deliver.

##### Container thermal control

The airflow, at high or low speeds, is driven by the fans through the evaporator. This evaporator needs to cool away: (1) heat produced by the evaporator fans and other equipment; (2) the heat loss due to air infiltration by fresh air intake; (3) the product heat load, namely the heat still stored in the product that needs to be cooled away in case the cargo was not entirely precooled to the supply air temperature; (4) the respiration heat from the fruit or vegetables; (5) the heat transmission losses through the container walls. Note that also latent heat is extracted from the air when moisture evaporates from the products. After the evaporator, a heater is in place. The heater has three functions, in order of importance:

1. Periodic defrosting of the evaporator coil at set points below approximately 5°C.
2. Avoid too cold supply air temperature during chilled transport.
3. Dehumidify the supply air in chilled mode shipments where a humidity set-point is used. Here, the heater is turned on and invokes a colder evaporator temperature, hence extra dehumidification.

**Fig. 5:**
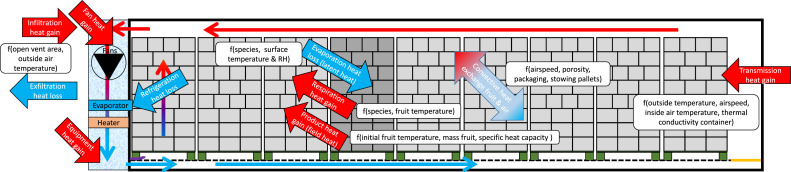
Heat loads in a container (side view). Red indicates a heat gain that needs to be cooled away, and blue indicates a heat loss or extraction from the container. One pallet is indicated in dark grey to illustrate which heat is extracted and gained from a pallet. The nomination f() is used to indicate to which parameters each heat load is a function of.

After passing the evaporator, the cold air is delivered into the cargo hold at the supply/delivery air temperature (T_sup_, T_DAT_). The entry is via an inlet slot at the bottom of the container. The air is driven from bottom to top through the cargo in a chilled model for respiring fresh fruit and vegetables, which are packed in ventilated packaging. For frozen cargo, the air is driven around the cargo. This cold air then absorbs the heat from the cargo and the heat entering the cargo from outside. This air is extracted via an outlet slot at the top of the container, back to the reefer unit, and the evaporator, where the return air temperature is measured (T_ret_). The difference in supply and return air temperature depends on the container's different heat loads (depicted in Fig. 5) and the rate at which the heat is removed from the fruit. The heat exchange rate depends on the airflow rate (so on the pressure loss over the pallets) and the air access to the ventilated cargo. This access depends on the ventilated packaging design, container stowing strategy, and airflow bypass.

##### Humidity control

**Fig. 6:**
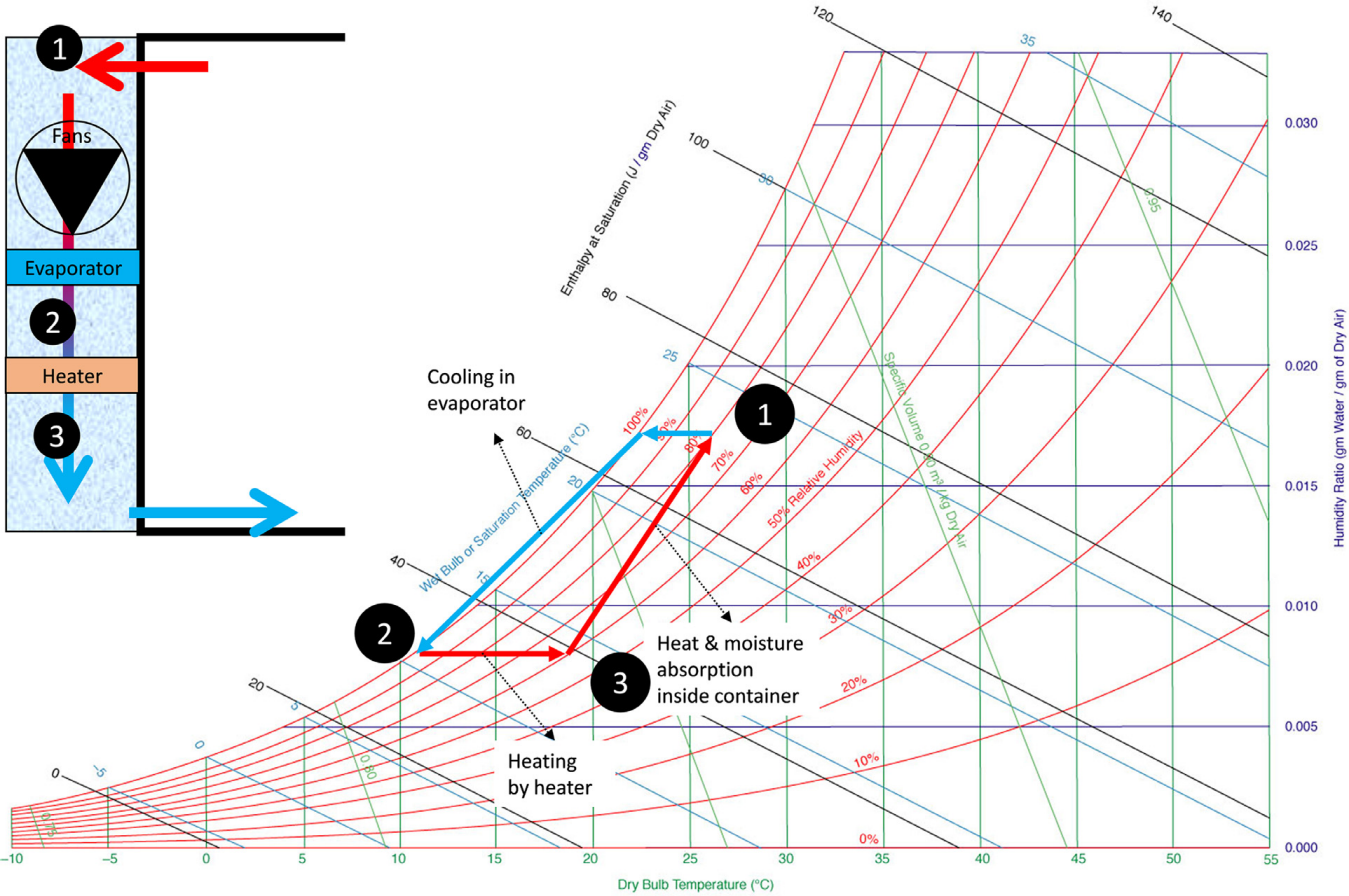
Psychrometric chart with an indication of the operation of a refrigerated container (adapted from [19]).

The air extracted from the container is sent to the evaporator unit. The reduction in temperature in this region increases the air's relative humidity, which typically can induce condensation or frost formation on the evaporator fins (Fig. 6). This process removes water vapor from the air and lowers the supply air temperature even to subzero temperatures. Cooling thereby dehumidifies. In a small number of shipments (typically garlic, onions, and flower bulbs), a humidity set-point of around 70 % is specified to invoke extra dehumidification. The heaters are turned on when the humidity set-point is specified, and humidity is above the set-point. The supply air temperature controller responds by adjusting the evaporator temperature. To further magnify the effect, the evaporator fan speed is often reduced. When the air enters the cargo hold again, water vapor in the air can increase due to the evaporation of fruit transpiration and previously-condensed liquid water. Also, the temperature of the air increases, by which the air can hold more water vapor as well. The higher the return air temperature, the more moisture can be stored in the air, so the more the evaporator can remove by cooling. The refrigeration unit typically leads to net dehumidification of the air and cargo. With fresh air intake, more moisture is introduced into the container. But almost all inlet moisture is immediately captured on the cold evaporator before it enters the cargo space, as the fresh air first passes the cold evaporator before entering the cargo space. The humidity in refrigerated containers typically equilibrates to 85–95 %.

##### Fresh air intake

Air intake is often set to remove metabolic gasses (CO_2_, C_2_H_4_). Still, it also leads to the intake of additional humidity [4] regulated by two openings in the container upstream and downstream of the evaporator fans (Fig. 3). The pressure difference over the fans draws in the fresh air and thereby only works efficiently when these fans are switched on. This fresh air is typically warmer and contains more water vapor. This air intake introduces additional moisture, which increases the need for defrosting. Furthermore, the warmer air can complicate temperature control. Typical air intake rates can be set from zero to about hundred m^3^ h^−1^ and are chosen depending on the commodity [4,7]. In the case of citrus, air intake rates are typically set to 15 m^3^ h^−1^.

##### Refrigeration unit & power consumption

**Fig. 7:**
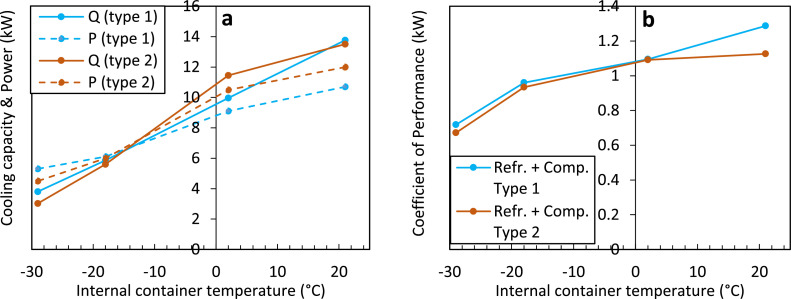
Net refrigeration capacity (Q_cool_), power consumption (P_cont_) (a), and resulting COP (b) of a refrigerated container for two types of compressors (Comp.) and refrigerants (Refr.) [6].

The refrigeration unit in the container has a refrigeration capacity [kW] that depends on the set temperature inside the container and the outside ambient temperature. The net refrigeration capacity is given in Fig. 7a. The resulting power consumption of the container is also shown. The higher the internal temperature in the container is set, the higher the required electrical power when running at its maximal refrigeration capacity, and the higher the refrigeration capacity [6]. Typically, a refrigerated container's required power is measured to be 2.7–3.6 kW per TEU (Twenty Foot Equivalent Unit, [6,20]), so about 5.4–7.2 kW for a 40-foot container. This is representative of the transport of products in chilled mode. Traditionally, transport in frozen mode used to require less power due to inefficient operation in the chilled range. The ratio of this net refrigeration capacity, so the heat removed from the container (Q_cool_) to the power required for the container to do so (P_cont_), is the coefficient of performance (COP [21]). The COP equals:

(3) $$COP=\frac{Q_{cool}}{P_{cont}}$$

The COP is, in this case, below 1.4 (Fig. 7b). Of this power consumption (P_cont_), the fans require about 1.7 kW at high speed and produce additional heat that needs to be cooled away [22]. The required power is supplied by clip-on generator sets ('gen-sets') during transport by road or rail and by the ship during marine shipping. These gen-sets deliver about 15 kW [4]. Note that the refrigeration unit needs to dissipate this heat that is cooled away from the container and the additional heat produced by the fans and other equipment into the environment. This amount depends on the type of respiring cargo loaded, the fan operating state, the operating state (chilled/frozen), and the corresponding internal and external temperatures. Typically between 7 and 15 kW [6] (Q_cool_) needs to be cooled away. This heat is then released into the environment surrounding the container. Such heat dissipation must be accounted for on ships, especially as many containers are packed in a tight space. Note that the numbers specified here on the power and energy use are subject to change as new containers with lower energy use are being developed and deployed.

##### Defrosting cycle

The evaporator cooler unit needs to be defrosted periodically. The reason is that frost builds up if surface temperatures in the evaporator are below zero. The frost on the evaporator reduces heat transfer performance from the evaporator to the air and induces additional airflow blockage. This higher airflow resistance, thereby, can change the operating point of the fans since the airspeeds through the evaporator are large. Frost formation often occurs if the cargo's set (delivery) air temperature is below 5–10 °C [6]. Such defrosting implies that the fans are stopped, and the evaporator unit and drip tray are heated electrically. Also, hot-gas defrosting of the evaporator is used by some manufacturers. Such defrosting cycles are noticed in the return air temperature data, where higher temperatures are logged due to the rising hot air from the heating. The control of the defrosting cycle is typically done at regular time intervals (e.g., 6h, 8h, 12h, [6]). Automatic defrosting control is also done at variable time intervals, depending upon need [7], which optimizes defrosting activity. Controllers can pick up the need for defrosting if the supply air temperature is not reached over an extended period or if the temperature difference between supply and return air is too high. The duration of the defrosting period, however, is variable. A defrost cycle is typically terminated when a defrost termination sensor, mounted just above the evaporator, reads a value above a certain treshold [6]. Once the threshold for end temperature is reached, the defrosting is stopped, and the fans are switched on again.

##### Remote container monitoring & management

A recent development in container monitoring is called remote container monitoring and management (RCM [7,[23], [24], [25], [26]]). Now, real-time information is available in the cloud on the set point, supply and return air temperature, humidity in the container, and machine operation (power-off periods and fan settings). For controlled atmosphere transport, also oxygen and carbon dioxide levels are monitored. The transfer of information from the container is done via a cellular network-enabled device on the refrigerated container that transmits data when connected and logs data when no connection is available. The key beneficiaries of RCM are owners of refrigerated containers or transport-service providers. They can mitigate with the use of RCM the risk of erroneous set-point adjustments and malfunctioning containers [25]. In addition, RCM increases the shipments' visibility and transparency and allows to replan shipment logistics in advance based on the hygrothermal history of the cargo. Notifications and alarms can be sent during transit, including unwanted door openings. For ambient loading, without precooling, the initial pulldown can be monitored. This is essential for banana transport and some citrus shipments [22]. RCM enables, in principle, in-transit adjustments of the set points for delivery air temperature and ventilation settings. However, despite this opportunity to adjust the set points in transit, tailoring such interventions by the stakeholders for each shipment requires the right metrics for decision-making. Stakeholders need to have detailed, real-time information on how these environmental conditions within the cargo (H_2_O, CO_2_, O_2_, C_2_H_4_) affect the fruit quality attributes, which is, to date, still challenging.

The data that is logged by the container via RCM can be made available to stakeholders, for example, importers. Note that each large refrigeration unit manufacturer often has its own (cloud-based) software to access these RCM data. However, a second line of monitoring of the hygrothermal conditions of the cargo is often done in parallel. One or more sensors are placed into the cargo, typically the last pallet near the door end, during loading. This sensor is placed, for example, by the company packaging and shipping the fruit. These sensor data are available via another (often cloud-based) platform and are used, for example, by retailers or importers, to verify the conditions during transport. One reason is that this is an independent system with which retailers can track all their shipments, independent of which refrigerated unit brand the container was equipped with. There are, thereby, for a container often two datasets that monitor the hygrothermal behavior of the cargo: one sensed by the refrigeration unit company and one sensed by independent sensors placed by the exporter. Interfaces that connect the two datasets, unfortunately, seem still missing or are scarce [24].

##### Cold disinfestation

Cold treatments are one of several effective phytosanitary risk mitigation measures required by an importing country. More recently, industries have started to move towards system approaches, which also include low-temperature shipping, but drastically reduce costs, carbon footprints, and food losses [27,28]. However, standalone in-transit cold disinfestation treatments are applied to eradicate live pests during the export voyage, such as false codling moth larvae or fruit fly larvae. For example, cold treatment protocols for false codling moth larvae (FCM) (*Lepidoptera: Tortricidae*)) and fruit flies (*Ceratitis capitata* and *C. dorsalis*) typically entail fruit first being precooled to target temperature before loading. Subsequently, supply air temperatures are set to ensure the fruits are maintained below 0 °C for 16 d during transport as a probit 9 disinfestation treatment [29,30]. Export to countries that do not require disinfestation treatments is normally done by precooling the fruit to about 4 to 7 °C before loading and shipping at the same temperature. Cold treatments represent a niche component of exports, as the treatment process is costly and substantially increases the risk and incidence of chilling injury to the fruit.

### Modeling airflow and heat transfer in porous media

#### Airflow and convective heat transfer in porous media

**Fig. 8:**
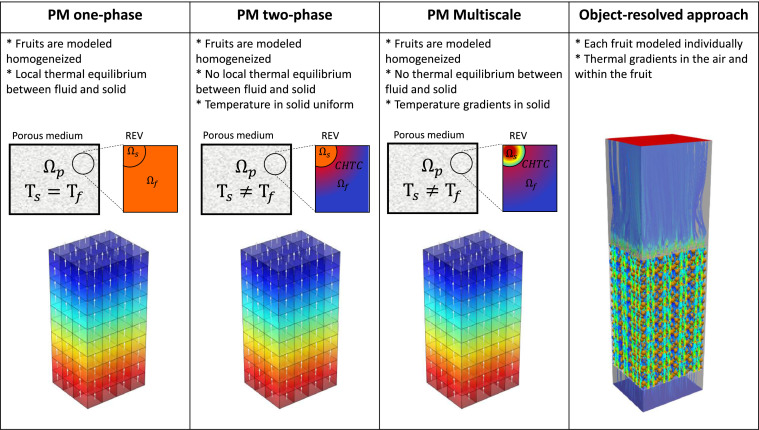
Different approaches to model heat and mass transfer in porous bulks of fresh horticultural produce (Ω is the domain type for porous medium, fluid or solid; CHTC is the convective heat transfer coefficient; REV is representative elementary volume).

There are four main approaches for physics-based modeling of airflow and the resulting convective heat transfer during refrigerated postharvest operations for fruit and vegetables (Fig. 8). They are listed in increasing order of complexity: porous medium modeling with the one-phase approach (PM one-phase), the two-phase approach (PM two-phase), multiscale porous-medium modeling (PM Multiscale), and the object-resolved approach.

##### Discrete, object-resolved approach

In the discrete, object-resolved approach, individual products and the heat and mass transport are modeled explicitly based on computational fluid dynamics (CFD). The Navier-Stokes equations for turbulent flow are solved in a computational domain, and every fruit is resolved explicitly. This detail captures the turbulent microclimate around each fruit and the thermal gradients within the fruit. The loss of momentum due to pressure drag and skin-friction drag is explicitly accounted for by solving the flow field and boundary layer around each fruit. This object-resolved methodology has been used in postharvest science to analyze a selected number of fruit in ventilated packaging in precooling processes [2,[31], [32], [33], [34]], refrigerated containers [[35], [36], [37], [38]] and cold storage at producers, wholesalers, retailers [39,40], among others. The focus was, among others, on improving uniformity in cooling within the shipment or optimizing ventilated packaging design. This high-resolution methodology is computationally costly, especially when modeling large quantities of products, for example, a refrigerated container containing over 100,000 products. To our best knowledge, the maximal amount of fruit that was modeled discretely was a full pallet containing 5000 fruit [38,41].

##### Porous medium approach

Alternatively, the less computationally-expensive porous medium approach can be used [42,43]. This lumped approach simulates the porous assembly of packed fruit and packaging materials without modeling each individual fruit. Instead, the fruit-carton-air system is represented as a porous medium. The transport equations for momentum and heat transport are adjusted accordingly. For momentum transport, volumetric sink terms are inserted in the Navier-Stokes equations to account for additional pressure losses from viscous, inertial, and near-wall confinement effects (Section 1.3.2). Convective heat or moisture transfer in the porous medium is tackled via the single-phase and two-phase approaches.

**Fig. 9:**
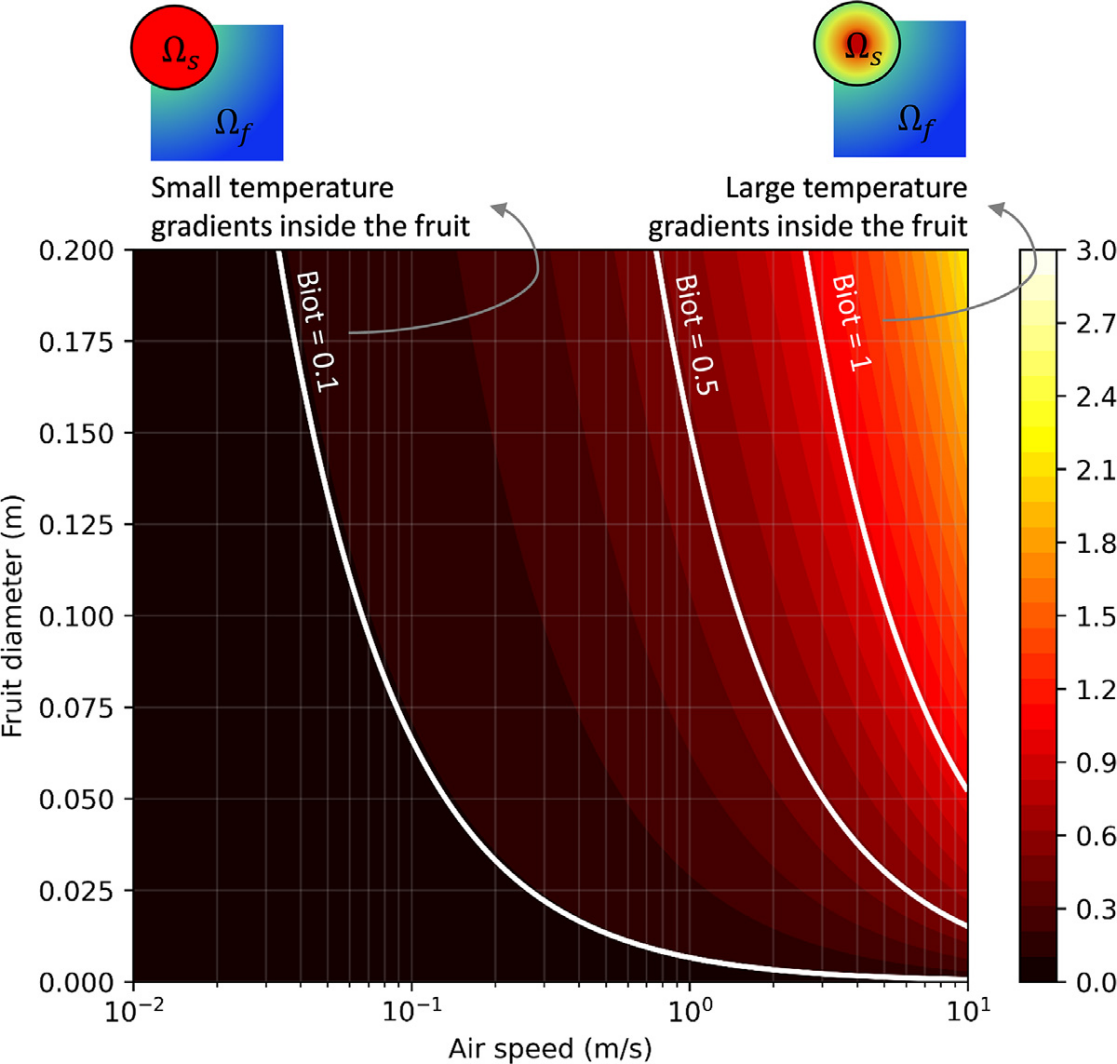
Biot number as a function of the fruit diameter and the approach flow airspeed for a single fruit for orange fruit. The convective heat transfer coefficient to calculate the Biot number is taken from Eq. (49) for a single sphere, so using a porosity of 1 in this equation.

The Biot number is an important parameter to evaluate which method can be accurately applied. This number is shown in Fig. 9 as a function of fruit diameter and airspeed for fruit with the thermal properties of an orange.

##### The Biot number

The Biot number is defined as the ratio of the convective heat transfer coefficient h_c_ [W m^−2^ K^−1^] and the characteristic length L_H_ [m] to the thermal conductivity of the solid λ_s_ [W m^−1^ K^−1^].

(4) $$Bi=\frac{h_{c}L_{H}}{\lambda_{s}}$$

The characteristic length for heat transfer L_H_ can be estimated as the ratio between the product's volume V [m^3^] and the surface area via which heat is exchanged with the environment A_sf_ [m^2^].

(5) $$L_{H}=\frac{V}{A_{sf}}$$

For a spherical product, this characteristic length can be written as a function of the particle radius r_p_ [m]:

(6) $$L_{H}=\frac{\frac{4}{3}\pi r_{p}^{3}}{4\pi r_{p}^{2}}=\frac{r_{p}}{3}$$

The characteristic length in the Biot number is thereby different from that of the Reynolds number, which is the diameter of the fruit.

##### Validity of PM approach and the REV

In principle, the porous medium approach models the fruit-packaging system as a continuum. This implies that each macroscopic point is assumed to be the center of a representative elementary volume (REV, [44]), where all these phases are superimposed on one another, implying that they cannot be distinguished separately. The porous material is thus modeled as a homogeneous material in which microscopic heterogeneities are averaged over each REV. These heterogeneities are, for example, vent holes or holes between the fruit. Transport inside the porous material is modeled by (effective) material properties, which are obtained experimentally or via numerical simulation at smaller scales. Examples are air permeability or thermal properties. The complex transport pathways and processes at the microscale level, so through the ventilated packaging, are included in a lumped way in the material transport properties and transport equations [44]. As such, the packaging and the fruit are not modeled discretely.

For this continuum representation of the porous medium to be theoretically valid, the REV size (L_REV_) should be sufficiently larger than the microscopic heterogeneities (e.g., the size of a pore or a vent hole, l_MIC_). This is essential for the averaging procedure of a certain quantity of interest, e.g., the density or porosity, to be accurate over that REV. In principle, the homogenization approach is not valid if the materials or phases are not homogeneous within a REV. If a REV is too small, it can fall fully within a pore or the solid material (e.g., fruit), by which the used homogenized properties are not valid there. In principle, the REV should also be smaller than the distance over which macroscopic variations in material properties (l_MAC_) are found. On the other hand, a REV should also be small enough to avoid variations of quantities within the REV (e.g., temperature) due to macroscopic gradients and associated non-equilibrium conditions at this microscale level. The latter is called the principle of separation of scales, namely l_MIC_ << l_REV_ << l_MAC_.

Since the continuum transport equations are solved on a computational mesh, the size of the computational cells with which the porous medium is meshed should typically be larger than the REV. If the mesh is smaller, then one mesh cell could include, for example, only part of a single fruit, by which the material properties in such a computational cell cannot be assumed to be homogenous.

In the case of palletized fruit in ventilated packaging, we face the problem that the micro and macro scales lie quite close to one another. The microscale would be the size of the fruit and the pores, so about 0.10 m. The macroscale would be the width or height of the pallets, so 1-2 m. The size of the REV should lie well between these two values: 0.1 m << l_REV_ << 1 m, which is difficult to achieve. In our case, the REV size and the mesh are typically not much larger than the pore size or vent holes. In some regions, the mesh can even be smaller to resolve airflow and heat transfer in a numerically stable way. It is well possible that these mesh elements that are smaller than the REV have no effect on the solution. Nevertheless, it can induce some differences, but this was not investigated to our best knowledge. However, it is computationally very demanding often to model the entire cargo with a discrete approach. Note that for very small fruits, such as berries, the size of the REV could be taken such that it satisfies better the separation of scales.

##### Single-phase approach

In the single-phase approach, the air and solid phases are lumped into a single phase. This approach assumes that both materials are well-mixed entities [11,45]. The effective thermal properties are derived via averaging, based on the relative amount of fruit versus air and the porosity. Local thermal equilibrium (LTE) between the gas and the solid phase (fruit) exists.

Thereby, all materials are always at the same temperature. Such an approach is, however, only realistic if the differences in thermal properties between the two materials are not very large and if the surface area for heat exchange is large, so if the diameter of the products is small [46]. In addition, local thermal equilibrium assumes that airspeeds are rather low so that equilibrium can set in. This situation is not the case for fruit stacked in a pallet and cooled with air, as there we have: (1) rather large particles compared to the flow path length, i.e., 3.7 % (= 0.08 m / 2.16 m); (2) non-negligible airspeed (e.g., 0.02–0.06 m s^−1^ for refrigerated transport); (3) a large difference between the thermal conductivity of air vs. (orange) fruit (λ_f_/λ_a_ = 22). Furthermore, the air phase will respond much faster to a temperature change than the fruit due to the large specific heat capacity of the fruit, compared to that of the air (c_p,f_/c_p,a_ ≈ 10^3^). Therefore, the single-phase approach is rarely used for this application and is also not viable. For example, it is more viable for hydro-cooling or when only natural convection is present. Finally, this approach also assumes that no thermal gradients are present within the fruit. This implies low Biot numbers, so low airspeed and/or small products.

##### Two-phase approach

The two-phase approach separately solves transport equations for the air and solid phases (Section 1.3.2). A convective transfer coefficient (h_c_ [W m^−2^ K^−1^]) accounts for the convective exchange at the air-product interface. In addition, the specific surface area for heat exchange between the fruit and the fluid needs to be specified (A_sf_ [m^2^ m^−3^]) (Section 1.3.2). The two phases are not necessarily at the same temperature, and a local thermal non-equilibrium (LTNE) is present.

However, the thermal gradients inside the fruit are still not accounted for with this approach since fruit size or shape is not explicitly included in the conservation equations. This is valid for low Biot numbers, so low airspeed and/or small products. However, as a workaround, the convective heat transfer coefficient and specific surface area for heat exchange can be artificially adjusted to account indirectly for fruit size and shape and, thus, thermal gradients inside the fruit.

Both porous medium approaches are powerful. However, their key limitation is that they do not account for thermal gradients inside the fruit. At high Biot numbers, such gradients occur during precooling or for very large fruit. In this case, a porous medium approach does not enable distinguishing between pulp and surface temperature. Workarounds have included adding an internal resistance in addition to the convective heat transfer coefficient to calculate the average temperature inside the fruit [43]. This resistance includes an equivalent penetration depth, dependent on the cooling speed. This limitation of not including the temperature gradients in the fruit hinders pinpointing critical events occurring at high Biot numbers. We do not know if the core and surface temperatures are different, by which quality differences can occur within the fruit. We cannot accurately identify sites where surface condensation on the fruit occurs. The pulp temperature is also not directly available since the kinetics of the cooling of individual fruit and the local microclimate is not captured, although being a key parameter to evaluate fruit quality.

##### Multiscale approach

Multiscale modeling is an alternative to the object-resolved approach that captures each fruit's thermal gradients while simultaneously capturing the entire shipment's thermal behavior. Multiscale modeling implies then modeling from the single-fruit-scale to the shipment scale. This method is not yet widespread in postharvest science, even though several multiscale modeling approaches exist for porous media [47]. Both one-way (sequential) and two-way (parallel, concurrent) coupling can be done between different scales [48]. Multiscale models in postharvest research have mainly focused on momentum transport, so fluid flow. Rarely heat and mass transport are considered. Fruit quality evolution within shipments is also not treated.

**Example of advanced multiscale model setup**. In postharvest refrigerated transport, a multiscale model could be built up as follows, among others. First, a two-phase porous medium model of the fruit in the shipment is built based on existing porous medium models (e.g. [42,43,49]). The fruit and packaging act as the porous medium's solid matrix, and (moist) air acts as the pore space. This macroscale model is extended to include relevant transport processes, for example: (1) loss of momentum via Darcy-Forchheimer-Brinkman; (2) turbulence generation and dissipation by both volumetric source and sink terms in the transport equations, namely for turbulent dissipation rate and turbulent kinetic energy; (3) convective heat and moisture transfer at the interface of the air with the fruit; (4) radiation scattering and absorption; (5) phase change (condensation, evaporation) based on the fruit's surface temperature. This macroscale model requires different model coefficients to calculate airflow, heat, and mass transport. Examples are viscous and inertial pressure loss coefficients, turbulence model coefficients, and convective transfer coefficients. These transport parameters can be separately determined empirically or by calculating the same transport processes on a representative elementary volume (REV) of a periodic microstructure, typically a small ensemble of fruit such as a carton. This REV model calculates these transport parameters based on packing density, open porosity, tortuosity, product size, etc. This information on the spatial variability of the environmental conditions throughout the entire cargo at the shipment scale is then used in a further downscaling step (localization) to calculate – concurrently – the hygrothermal state and resulting key quality attributes (KQA) of every single fruit in each REV. This step is essential to capture internal gradients within each fruit. The macroscale and microscale simulations run simultaneously with their internal time steps. For example, the information exchange between the models can be done via a temporal coupling using an explicit (staggered) coupling procedure. This coupling is done at predefined points in time, namely well below the typical time scales for heat transfer or quality decay within the fruit. Smaller time steps are required with an explicit coupling compared to implicit coupling, but an explicit coupling is preferred for its enhanced stability.

##### Alternative approaches

Hybrid approaches have also been used, where ventilated packaging and its ventilation openings have been modeled discretely, but the fruit inside these packages is modeled as a porous medium [50]. As mentioned above, the separation of scales is not guaranteed in our case since the size of the REV in the porous medium, and so the mesh is of the same size as the vent holes and pores between the fruit. As such, using the hybrid approach can also induce some differences with a discrete approach, but this needs to be investigated.

##### Approach used in this study

**Fig. 10:**
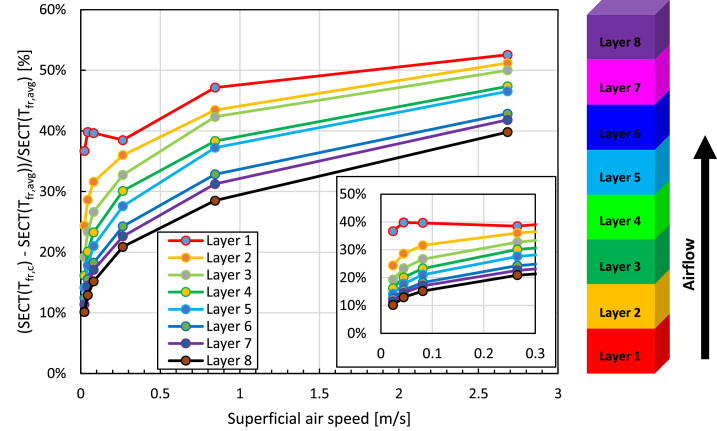
The relative difference in the seven-eighths cooling time, calculated based on fruit core temperature and average fruit temperature at different airspeeds for different layers of cartons in a pallet for vertical airflow through a stack of palletized boxes (data from [36]).

This study focuses on cooling fruit on pallets inside a refrigerated container. The maximal possible airspeeds in a container are: (1) a superficial airspeed of 0.048 m/s, assuming that all air (4150 m^3^ h^−1^) flows through the 20 pallets and no airflow bypass is present; (2) a corresponding physical airspeed of 0.126 m s^−1^, assuming a porosity of 38 %. Note that locally higher airspeeds can occur near the vent holes. At these airspeeds, the Biot number will be below about 0.12 (Fig. 9) for the fruit of a diameter of 80 mm when calculating the CHTC according to Eq. (52). This Biot number implies the thermal gradients within the fruit will be rather limited. As such, we use the two-phase approach in this study. This implies that both the fruit and the air are modeled thermally separate, so the particles in the porous medium and the pores have different thermal properties and, thereby, also react thermally at a different speed. In a previous study [36], we already identified rather limited differences between fruit average and core temperatures for this airspeed range. As an illustration, we compared the resulting SECT in Fig. 10, calculated using core and average fruit temperature. We get rather small differences below 20 %, except for the cartons on the bottom of the pallet. These differences become larger at higher airspeeds, so higher Biot numbers.

#### Momentum loss in porous media

We model the momentum loss in porous media by using the Darcy-Forchheimer-Brinkman equation. This equation relates the pressure gradient over the porous medium (∇p[Pa m^−1^]) to the velocity (**u** [m s^−1^]). This equation originates from Darcy's law.

Darcy's law is derived from the Navier-Stokes equations, which is simplified to the Stokes equation by assuming stationary, incompressible, and creeping flow without any body force. When it is assumed the viscous resistance increases linearly with the velocity and an isotropic porous medium is present, Darcy's law is obtained.

(7) $$q=\frac{Q}{A}=-\frac{k}{\mu }\nabla p$$

Here q is the volumetric flux [m s^−1^], Q is the volumetric flow rate [m^3^ s^−1^], A is the surface area [m^2^], μ is the dynamic viscosity of the fluid [Pa s] [kg m^−1^ s^−1^] and k is the permeability [m^2^].

The Darcy-Forchheimer-Brinkman equation is [13,51,52]:

(8) $$\nabla p=-\frac{\mu_{a}}{k}u-\frac{c_{F}\rho_{a}}{k^{1/2}}u\left|u\right|+\mu_{a,eff}\nabla^{2}u=C_{Da}u+C_{Fo}\rho_{a}u\left|u\right|+C_{Br}\nabla^{2}u$$

(9) $$\begin{array}{c} C_{Da}=-\frac{\mu_{a}}{k}\\ C_{Fo}=-\frac{c_{F}}{k^{1/2}}\\ C_{Br}=\mu_{a,eff} \end{array}$$

Here, p is the pressure [Pa], μ_a_ is the dynamic viscosity of the fluid, which is air in this case [Pa s] or [kg m^−1^ s^−1^], k is the Darcy permeability [m^2^], c_F_ is a dimensionless Forchheimer coefficient [-], ρ_a_ is the fluid density [kg m^−3^], μ_a,eff_ [13] is the effective viscosity (μ_a,eff_ = μ_a_τ/ε), and **u** is the average air velocity. Sometimes an alternative effective viscosity is used in the equations, namely μ_a,e_ [53] (μ_a,e_ = μ_a_ τ = ε μ_a,eff_). C_Da_, C_Fo,_ and C_Br_ are the Darcy, Forchheimer, and Brinkman loss coefficients.

The mentioned velocity is the average velocity within the porous medium volume. This includes both solid matrix and pore space. This air velocity and the corresponding airspeed are called superficial (discharge) velocity **u** and speed U [m s^−1^]. The physical (intrinsic, seepage) velocity **v** or speed *V* in the pore space is higher and equals **v** = **u/**ε (or **u** = **v**.ε), where ε is the porosity [13,52]. When substituting the superficial velocity **u** with the physical velocity **v**, we get:

(10) $$\begin{array}{c} \nabla p=-\frac{\mu_{a}}{k}\varepsilon v-\frac{c_{F}\rho_{a}}{k^{1/2}}\varepsilon^{2}v\left|v\right|+\varepsilon \mu_{a,eff}\nabla^{2}v\\ \nabla p=-\frac{\mu_{a}}{k}\varepsilon v-\frac{c_{F}\rho_{a}}{k^{1/2}}\varepsilon^{2}v\left|v\right|+\mu_{a,e}\nabla^{2}v \end{array}$$

##### Darcy-Forchheimer-Brinkman terms

In this equation, the first term (Darcy) on the right-hand side represents pressure loss due to viscous forces, which increases linearly with the velocity. The second term (Forchheimer) represents pressure loss due to inertial forces occurring in the flow, which increases quadratically with the velocity. The Forchheimer term does not imply turbulent flow, however. The third term (Brinkman) represents pressure loss due to viscous forces occurring in near-wall flow due to confinement, which increases linearly with the velocity. Each of these terms is only significant under certain conditions. Darcy dominates under low-speed conditions, whereas Forchheimer dominates at high speeds. The Brinkman term is only relevant close to the exterior walls or surfaces with which the porous medium is in contact (via the divergence of the velocity gradient), as only at these locations are velocity gradients present.

##### Particle Reynolds number

The particle Reynolds number (Re_p_) can be determined based on the approach flow airspeed around the particle and a characteristic length for fluid flow L_F_ [m], for which the particle diameter (*d_p_*) is typically chosen. This characteristic length is representative of flow around the sphere, for example, the flow structures such as vortices that are generated downstream of a sphere. The approach flow airspeed in the pore space is the physical speed V in the porous medium, so around the particles. The Reynolds number can be written as:

(11) $${\text{Re}}_{p}=\rho \frac{V.d_{p}}{\mu }=\frac{U.d_{p}}{\varepsilon \nu }$$

This definition is in line with the typical definition of the Reynolds number. Previous work on flow in packed beds also used this [54,55]. This Reynolds number increases if the porosity decreases for a certain approach flow (superficial) airspeed U. The reason is that the physical airspeed to which the particles are subjected to, increases. This characteristic length for fluid flow (d_p_) differs from that of the Biot number for heat transfer, which was d_p_/6 (Eq. (6)).

Note that other authors used an alternative definition, which was derived differently for packed beds [13,56]:

(12) $${\text{Re}}_{p}=\rho \frac{U.d_{p}}{\mu \left(1-\varepsilon \right)}=\frac{U.d_{p}}{\nu \left(1-\varepsilon \right)}$$

Here the Reynolds number decreases if the porosity decreases, which seems counter-intuitive as the physical airspeed increases with decreasing porosity. This Reynolds number is used to characterize the flow regime, where Re_p_ lower than 1–10 are required to have purely Darcy flow [52].

##### Sphere packing density

The highest sphere/solid packing density (θ_s_=1-ε) that one can achieve for equally-sized spheres is 74 % so a porosity of 26 %. Irregular sphere packaging of equally-sized spheres leads to a maximal packing density of 64 % so a porosity of 36 %. The minimal porosity of a porous ensemble of equally sized spheres is thus higher than 26 %. A simple cubic lattice, where spheres of a diameter *D* are stacked on top of one another, has a porosity and solidity of:

(13) $$\begin{array}{c} \phi =\frac{V_{a}}{V_{tot}}=\frac{V_{tot}-V_{sphere}}{V_{tot}}=\frac{D^{3}-\frac{4}{3}\pi {\left(\frac{D}{2}\right)}^{3}}{D^{3}}=1-\frac{4}{3}\pi {\left(\frac{1}{2}\right)}^{3}=1-\frac{1}{6}\pi =48\%\\ \theta_{s}=1-\phi =52\% \end{array}$$

Here V_tot_ is the total volume [m^3^], V_sphere_ is the volume taken up by the sphere, V_a_ is the volume taken up by the air.

##### Ergun equations

The permeability *k*, Forchheimer coefficient c_F_ and the effective viscosity μ_a,eff_ depend on several factors, including the pore size, pore geometry, porosity, and tortuosity of the porous material. Here μ_a,eff_ (μ_a,eff_ = μ_a_τ/ε) is often simplified to μ_a_,_e_/ε, so neglecting any tortuosity effects. The permeability k and Forchheimer coefficient c_F_ can be determined via analytical/empirical formulations. A typical example is the Ergun equations for spherical particles [13,57]:

(14) $$\begin{array}{c} \frac{1}{k}=\frac{K_{1}{\left(1-\varepsilon \right)}^{2}}{d_{p}^{2}\varepsilon^{3}}\\ c_{F}=\frac{K_{2}}{K_{1}^{1/2}\varepsilon^{3/2}} \end{array}$$

*K_1_* and *K_2_* are constants equal to 150 and 1.75 for randomly stacked spheres, respectively, although also values of 180 and 1.8 have been reported [13]. Here, d_p_ [m] is the particle diameter. This diameter is straightforward to determine for spherical particles, namely twice the particle radius r_p_. For spherical-like particles, the equivalent particle diameter can be calculated as 6V_p_/A_p_, where V_p_ [m^3^] and A_p_ [m^2^] are the particle volume and surface area. This equation reduces to 2r_p_ for a spherical particle:

(15) $$6\frac{V_{p}}{A_{p}}=6\frac{\frac{4}{3}\pi r_{p}^{3}}{4\pi r_{p}^{2}}=2r_{p}$$

When neglecting the Brinkmann term, the pressure gradient and pressure loss over a certain distance L can be written as:

(16) $$\begin{array}{c} \nabla p=-\frac{\mu_{a}}{k}u-\frac{c_{F}\rho_{a}}{k^{1/2}}u\left|u\right|\\ \nabla p=-\mu_{a}\frac{K_{1}{\left(1-\varepsilon \right)}^{2}}{d_{p}^{2}\varepsilon^{3}}u-{\left(\frac{K_{1}{\left(1-\varepsilon \right)}^{2}}{d_{p}^{2}\varepsilon^{3}}\right)}^{1/2}\frac{K_{2}}{K_{1}^{1/2}\varepsilon^{3/2}}\rho_{a}u\left|u\right|\\ \nabla p=-\mu_{a}\frac{K_{1}{\left(1-\varepsilon \right)}^{2}}{d_{p}^{2}\varepsilon^{3}}u-\left(1-\varepsilon \right)\frac{K_{2}}{d_{p}\varepsilon^{3}}\rho_{a}u\left|u\right|\\ \Delta p=\frac{150\mu_{a}{\left(1-\varepsilon \right)}^{2}}{d_{p}^{2}\varepsilon^{3}}Lu+\frac{1.75\rho_{a}\left(1-\varepsilon \right)}{d_{p}\varepsilon^{3}}Lu\left|u\right|\\ \Delta p=C_{Da}Lu+C_{Fo}Lu\left|u\right| \end{array}$$

In postharvest problems, where ventilated and non-ventilated packaging is included (Fig. 1), such analytical formulations for momentum loss over stacks of spheres are less realistic. The reason is that in addition to the fruit size, shape, and stacking within the packaging, the pressure loss is also dependent on the packaging design (vent hole size, shape, and placement) and the stacking pattern of the packages on the pallet. The packaging often introduces a large pressure drop over the limited ventilated openings. Therefore, k and c_F_ for the composite system of packed fruit or vegetables are typically estimated experimentally or via physics-based simulations by quantifying the pressure drop over the porous medium at a certain flow rate or vice-versa [12,58,59].

##### Darcy versus Forchheimer for porous media

**Fig. 11:**
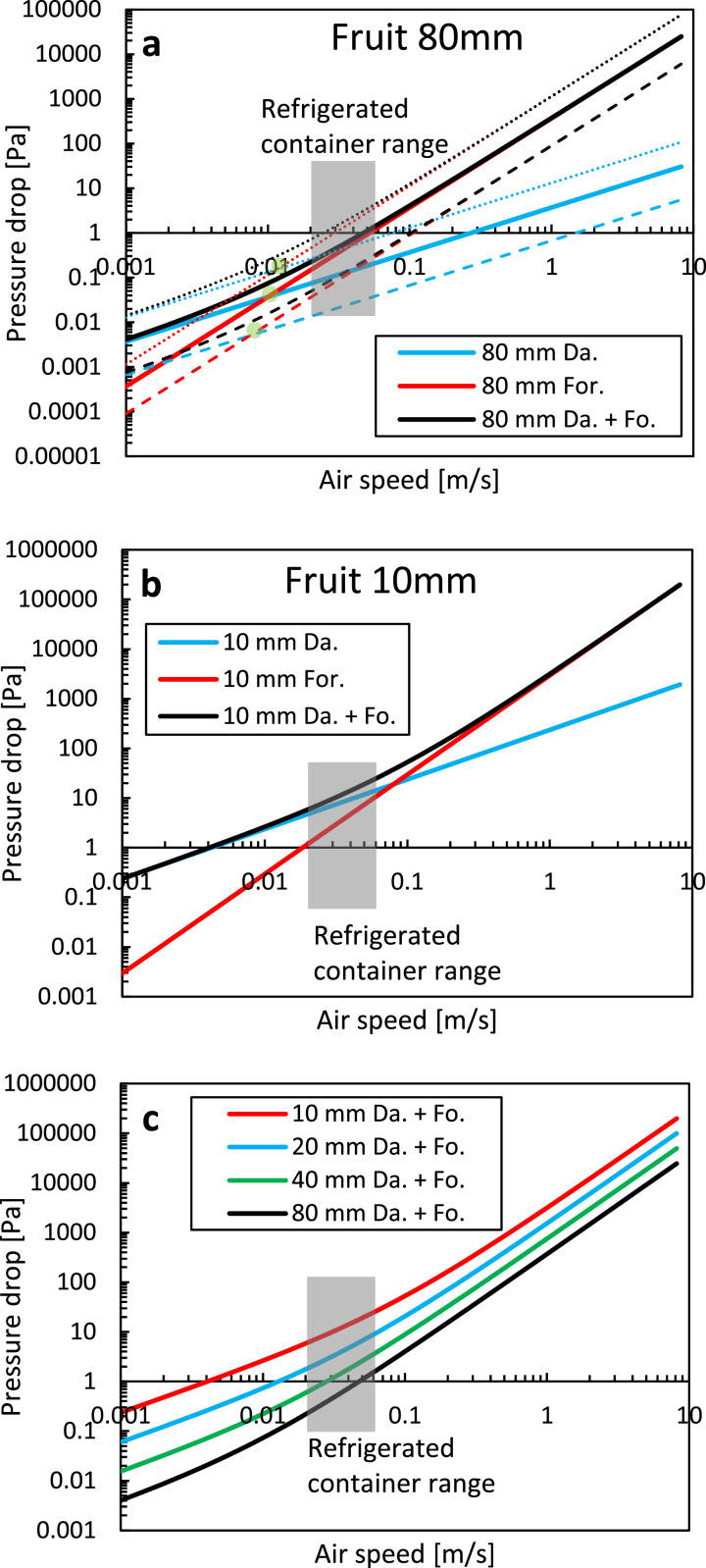
(a, b) Pressure loss as a function of airspeed, for the Darcy and Forchheimer terms, and both for two fruit sizes. In (a) also, the results at a lower (26 %) and higher porosity (52 %) are depicted with a dotted and dashed line, respectively. (c) Both terms together are depicted for four fruit sizes. The superficial airspeed range in a refrigerated container is also depicted by the grey area.

We quantify Darcy and Forchheimer's relative contribution to the pressure gradient (Eq. (8) or Δp/L). We calculate the pressure loss over a unit length (L) of 1 m. We quantified each contribution for equally sized spheres of different diameters with irregular packing. We relied on the Ergun equations (Eqs. (14), (16)), so a stack of spheres without the cardboard packaging. We assumed the following: (1) a porosity of 36 %, which is the minimal porosity that can be achieved for an irregular packing of spheres, (2) a fruit diameter of 80 mm and 10 mm (e.g., orange fruit vs. mandarin fruit); (3) The results are shown in Fig. 11.

The dependency of the pressure loss (Δp) on the Darcy and Forchheimer terms is as follows: (1) The larger the particle size becomes, the more dominant the Forchheimer term (proportional to 1/d_p_) becomes at a particular speed compared to the Darcy term (proportional to 1/d_p_^2^); (2) The larger the porosity becomes, the more dominant the Forchheimer term (proportional to (1-ε)) becomes at a particular speed compared to the Darcy term (proportional to (1-ε) ^2^).

**Fig. 12:**
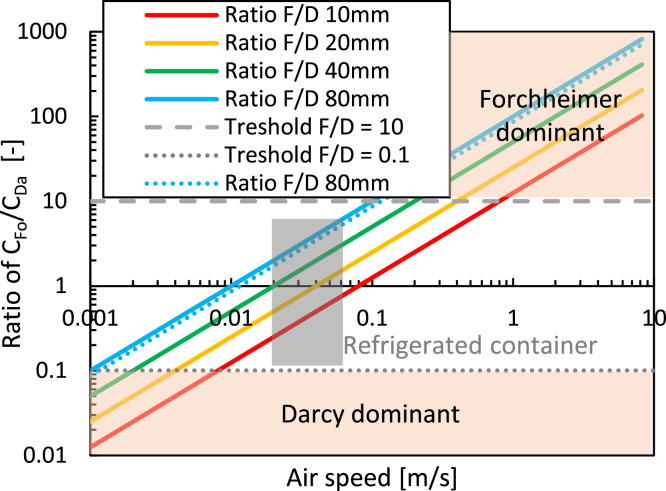
The ratio of Forchheimer to Darcy coefficient as a function of the airspeed for different particle sizes at a certain porosity. Also, the results at a lower porosity (26 %) for a sphere of 80 mm are depicted with a dotted line.

The main conclusions can be made for high packing densities, so low porosities. The Forchheimer term must always be incorporated for airflow in refrigerated containers (Fig. 12). For very small particles, the Darcy term only overshadows the Forchheimer term (i.e., C_Fo_ < 0.1 C_Da_) at airspeeds of 0.01 m/s, which is already rather low. Darcy flow is mainly dominant, compared to the inertial term, when the particle Reynolds number is below 1–10 [52]. Even then, C_Fo_ still plays a role. The critical velocity below which the Forchheimer term becomes negligible (C_Fo_ < 0.01 C_Da_) is 0.001 m s^−1^ for small fruit, which is very low, and higher airspeeds are always found with natural convection.

However, the Darcy term cannot be neglected, especially for airspeeds in refrigerated containers (0.01–0.1 m/s). For large fruit, the Forchheimer dominates the Darcy term (i.e., C_Fo_ > 10 C_Da_) at airspeeds of 0.1 m s^−1^. The Darcy term can, however, only be neglected (C_Fo_/C_Da_ > 100) at airspeeds above 1–10 m/s and at particle Reynolds numbers above about 10,000.

However, even for very densely packed stacks of spherical products (with regular or irregular packing, so a porosity of 26 %–36 %), the Darcy term is often less important for standard cooling operations (refrigerated container and precooling). Even in the worst case, where the Darcy term has the most impact (low porosities 36 %, small particle sizes 0.01 m), both terms are of equal size at a speed of about 0.1 m/s.

### Multiphysics continuum model

#### Computational system configuration

**Fig. 13:**
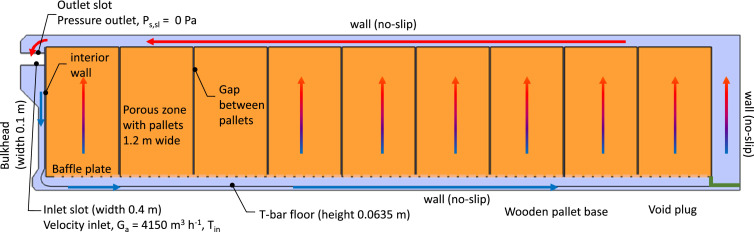
Computational model and boundary conditions.

A 2D computational model of the refrigerated container was constructed to study the cooling of citrus fruit during refrigerated transport. The model mimics the cooling of citrus fruit by vertical airflow for fruit packed in Supervent cartons and stacked on 20 pallets in a refrigerated container. This (base) model is shown in Fig. 13 with its dimensions and boundary conditions, where the latter is detailed in Section 1.3.6.

A two-dimensional model was chosen in this study to model airflow and heat transfer in the container instead of a three-dimensional model. A key reason for choosing a two-dimensional model is that we can run the model in an acceptable time (∼ hours) on a standard desktop computer or laptop. This feature is essential if we would like to widely disseminate and use this model. Our starting point is that currently, our stakeholders have only temperature sensor data. We can upcycle these sensor data with a physics-based virtual container by turning them into actionable metrics for the entire cargo. From this viewpoint, we transform the zero-(spatial) dimensional temperature data to two-dimensional spatial information inside the container, which is a huge step forward. Running our application is impossible for 3D simulations of millions of control volumes as, apart from computational time, the RAM is also lacking on standard computers. Airflow in a container is inherently three-dimensional, and most previous studies looked at three-dimensional container geometries. However, a two-dimensional representation can realistically approximate the 3D container for the following reasons:

- The inlet and outlet slots span almost the full width of the container, creating a quasi-2D inlet and outlet flow conditions.
- The height of the zones where air flows, excluding the porous pallets, is small compared to the container's width (2.29 m). These cavities include the bottom cavity (T-bar floor and the pallet base height = 0.224 m), the void at the door end of the container (width = 0.56–0.76 m, depending on the pallet row), and the top cavity above the pallets (height = 0.197 m). This leads to container width (W_co_) to cavity height (H_cav_) ratios of 10.2, 3-4, and 11.6, respectively (W_co_/H_cav_). The minimal aspect ratio between a channel's width and height to have a two-dimensional flow is 7:1 [60]. Thus, flow in the enclosures in a container can be considered two-dimensional, except for the void at the door end of the container. This implies that airflow in the inlet and outlet cavities is two-dimensional. In the void at the door end of the container, the side edges could lead to a somewhat different 3D airflow field prediction.

Simplifying the computational model with a 2D representation has a significant advantage. The simulations can be run much faster due to the much lower control volumes required. The mesh size is reduced by an estimated order of magnitude 10^2^. This reduction also proportionally reduces the computational time. Another advantage is that the stability of the simulation can be improved by highly refining the grid near the inlet. A fine grid is required since a large airspeed reduction into the T-bar floor occurs here, which can introduce numerical instabilities. As in previous studies, a 3D grid would require millions of finite elements to model the airflow realistically. The main disadvantage of the two-dimensional model is that lateral heat exchange with the outside environment cannot be directly included in the model as the side walls are not modeled. In addition, the impact of asymmetric stowage patterns and chimneys between four pallets (windmill configuration) can also not be studied. These losses are therefore included equivalently in the 2D model via the top, bottom, and vertical door end walls.

The computational model contains the following components. We describe each component indicated in Fig. 13 sequentially, based on the air's path. We discuss the model simplifications that are made to model the component:

- The inlet section, where the airflow produced by the evaporator fans, enters the container via the inlet slot. Here, our model included the entire supply air duct, starting from the outlet of the fans. This supply air duct in a refrigerated container was modeled similarly to [61]. This duct stretches along the entire container width, so it can be represented in 2D as well. The dimensions of the inlet duct were extracted from [61], images, and CAD drawings of refrigerated containers since the actual dimensions are typically proprietary information. In our study, the 2D-inlet duct includes a 400 mm wide section where the fans are located, a converging nozzle part that reduces the cross-section to 100 mm, a straight bulkhead section of 100 mm wide, and a corner section with a baffle plate that guides the airflow into the narrow channels of the container floor. Note that no components, such as the evaporator or heater after the fans, are included in the duct, although they are present in reality and will induce a large pressure loss. As such, the total pressure loss over the model will be lower than in reality.
- A baffle plate at the refrigerator unit side. The pallet base of the first pallets is usually placed on the baffle plate. The dimensions and shape of the baffle plate were based on those of a commercially-available baffle plate ([62], model 1311–8598).
- The narrow open space of channels within the T-bar floor (H_tb_ = 63.5 mm), where the air is directed horizontally and upwards.
- The orifices/openings in the T-bar floor, which induce a speed-up of the flow when exiting the bottom floor channel and a pressure loss. These openings lead to an open area (or porosity) of the T-bar floor of 55 %. The openings in the T-bar floor are explicitly modeled in the 2D computational model, which means we include each T-bar plate separately. However, the channels normally have an opening that stretches out over the full length of the container (x-direction). In our 2D model, we needed to model them as stretching out in the y-direction. Note that the thickness of the T-bars was not modeled to simplify the model and the computational mesh.
- The base of the wooden pallets, which rests on the T-bar floor. The wooden slats cover 39 % of the surface area of each pallet and thereby block a significant part of the airflow from the T-bar floor. The T-bar floor blocks 45 % of the floor area, and a significant overlap occurs with the wooden pallet bases. The pallet bases are not modeled explicitly, as the blockage by the T-bar floor already accounts for some blockage. It is difficult to realistically estimate or model how this blockage is translated in the 2D model.
- The narrow open space between the wooden pallets' base and the pallet's top wooden slats. Here only wooden connectors between the bottom and top of the base are present, which take up about 10 % of the volume in this space. These blocks are not explicitly included in the model.
- The top of the wooden pallet, where the wooden slats cover 76 % of the surface area of each pallet and thereby block a significant part of the airflow coming out of the T-bar floor. The pallet top wooden slats are modeled explicitly, namely, 9 slats of 102 mm wide per pallet. Since we have a 2D model, equivalent slat width and gaps between the slats (36 mm) were used to attain the same open area as in 3D, namely 24 %.
- The fruit that is palletized in cardboard cartons on the wooden pallet. For airflow, the porous-medium approach is used to represent the fruit palletized in carton boxes. Each of the 20 pallets is represented as a homogeneous entity containing 80 cartons and 5120 fruit. The porous media model represents 102,400 fruit packed in 1600 cartons. In total, 9 pallets are modeled in the 2D model. The width of the 9 palletized fruit (y-direction 1.2 m wide) is corrected to 1.212 m to account for the other row of 11 pallets in the container (y-direction 1 m wide). That way, the total volume of fruit in a full container in the 2D model is equivalent to the 3D model. The cartons were not included in the model for the thermal calculations, and only the fruit was considered. The carton has a much lower volume and mass than the fruit and, thereby, also a lower thermal storage capacity than the fruit (m x c_p_ [J K^−1^]), only 1.5 % of that of the fruit. Furthermore, heat transfer from individual fruit was governed by convection to the air, not by conduction to other fruit and then to the carton. As such, the carton's insulating effect is less critical.
- Small vertical gaps between the pallets of 20 mm are accounted for in the base model, by modeling them discretely. This value is based on observations in practice. Still, large variations in actual gaps between the pallets can occur, depending on the level of detail given when stacking the cartons on the pallet and when loading. Note that other studies have modeled the gaps as a porous medium [63]. Since a 2D model is used, these gaps, to some extent, also account for the lateral gaps between the two pallet rows.
- The open space above the pallets was modeled as an air gap.
- The return air duct, where the air from the cargo hold flows back to the refrigeration unit of the container. Similar to the inlet duct, the dimensions were also based on [61], drawings and images. The outlet of the computational domain is assumed to be the inlet of the fans. As such, the only component not included in the airflow model is the fans.
- The container walls are not explicitly included in the model. The thermal inertia of the walls is not accounted for. The heat that can be stored in the container walls (∼ m x c_p_ [J/K]) is only a small part (4 %) of the heat stored in the fruit. The field heat in the product comprises the main heat load. As such, this assumption will not largely affect the cooling behavior of ambient-loaded cargo.
- The transmission heat exchange of the container with the exterior environment is modeled. These transmission losses are about 1.5–2.6 kW [6,22]. The total heat flow that enters the container from the outside equals Q_trans_ = U.A_co,tot_(T_out_-T_in_), where U is the thermal transmittance [W m^−2^ K^−1^], A_co,tot_ is the total exposed surface area of the container to the outside [m^2^]. This surface includes the top and bottom walls, the side walls, and the vertical wall at the door end. The vertical wall at the refrigeration unit end is not accounted for, as the refrigeration unit is placed there. T_out_ and T_in_ are the outside and inside air temperatures, respectively. A refrigerated container is well insulated with a theoretical U ≈ 0.2–0.3 W m^−2^ K^−1^ or U.A_co,tot_ ≈ 40 W K^−1^) [14,22]. In practice, measured values are often a bit higher (U ≈ 0.3–0.4 W m^−2^ K^−1^ or U.A_co,tot_ ≈ 60 W K^−1^). We used a U.A_co,tot_ value of 42 W K^−1,^ and recalculated an equivalent 2D U-value to use in the simulations. In the 2D simulations, only transmission heat losses occur via the 11.56 m long roof, floor, and 2.58 m high wall at the door end over a width of 2.29 m. We obtain an equivalent U-value of 0.71 W m^−2^ K^−1^ for the 2D simulation.
- A void plug is installed at the back of the container to avoid airflow bypass via the back opening of the container. Airflow bypass would induce lower flow rates through the pallets and slower cargo cooling.

The simplifications above were made to keep the model complexity and computational cost within limits. These simplifications render the computational model into a more idealized case.

#### Governing equations for porous media modeling

##### Conservation equation for momentum in air and porous media

The conservation of momentum in the air and the porous medium are modeled by solving the Navier-Stokes equations. We assume incompressible dry air flow, with a constant density and viscosity, so quasi-isothermal conditions or small temperature differences. Solving these equations in the porous medium (Ω_PM_) requires that additional sink terms be accounted for in the porous medium and that the effect of the porosity on the airflow is accounted for.

(17) $$\begin{array}{c} \rho_{a}\frac{\partial w}{\partial t}+\rho_{a}w\cdot \nabla w=-\nabla p+\mu_{a}\nabla^{2}w+\chi_{\Omega_{PM}}S_{m,\mathrm{PM}}\\ \rho_{a}\frac{\partial w}{\partial t}+\rho_{a}w\cdot \nabla w=-\nabla p+\mu_{a}\nabla^{2}w+\chi_{\Omega_{PM}}\left(-\frac{\mu_{a}}{k}u-\frac{c_{F}\rho_{a}}{k^{1/2}}u\left|u\right|+\mu_{a,eff}\nabla^{2}u\right) \end{array}$$

(18) $$\chi_{\Omega_{PM}}=\left\{ \begin{array}{c} 1\text{in}\Omega_{PM}\\ 0\text{in}\Omega_{f} \end{array}\right.w=\left\{ \begin{array}{c} v\text{in}\Omega_{PM}\\ u\text{in}\Omega_{f} \end{array}\right.$$

Here, ρ is the air density (1.29 kg m^−3^ at 0°C), t is the time [s], μ_a_ is the dynamic viscosity of air, and **u** is the superficial velocity [m s^−1^]. The impact of the porous medium on the fluid flow is accounted for by including a volumetric sink term for momentum in the porous medium S_m,PM_ ([Pa m^−1^] or [kg m^−2^ s^−2^]) in the momentum equation. This source term equals the Darcy-Forchheimer-Brinkman equation (Eq. (8) [52]). $\Omega_{PM}$ and $\Omega_{f}$ stand respectively for the porous media and free fluid domains. $\chi_{\Omega_{PM}}$ equals 1 in the porous media and 0 in the free domain. The variable **w** corresponds to the interstitial/physical velocity **v** (**v** = **u**/ε) in the porous medium $\Omega_{PM}$ and to the superficial velocity **u** in the fluid domain $\Omega_{f}$.

In the porous medium, we rewrite this equation as a function of the superficial velocity or the physical velocity:

(19) $$\begin{array}{c} \rho_{a}\frac{\partial v}{\partial t}+\rho_{a}v\cdot \nabla v=-\nabla p+\mu_{a}\nabla^{2}v-\frac{\mu }{k}u-\frac{c_{F}\rho }{k^{1/2}}u\left|u\right|+\mu_{eff}\nabla^{2}u\\ \frac{1}{\varepsilon }\rho_{a}\frac{\partial u}{\partial t}+\frac{1}{\varepsilon^{2}}\rho_{a}u\cdot \nabla u=-\nabla p+\frac{1}{\varepsilon }\mu_{a}\nabla^{2}u-\frac{\mu_{a}}{k}u-\frac{c_{F}\rho_{a}}{k^{1/2}}u\left|u\right|+\mu_{a,eff}\nabla^{2}u \end{array}$$

When neglecting the Brinkman term, we get the equation used in this study:

(20) $$\begin{array}{c} \rho_{a}\frac{\partial v}{\partial t}+\rho_{a}v\cdot \nabla v=-\nabla p+\mu_{a}\nabla^{2}v-\frac{\mu_{a}}{k}u-\frac{c_{F}\rho_{a}}{k^{1/2}}u\left|u\right|\\ \frac{1}{\varepsilon }\rho_{a}\frac{\partial u}{\partial t}+\frac{1}{\varepsilon^{2}}\rho_{a}u\cdot \nabla u=-\nabla p+\frac{1}{\varepsilon }\mu_{a}\nabla^{2}u-\frac{\mu_{a}}{k}u-\frac{c_{F}\rho_{a}}{k^{1/2}}u\left|u\right| \end{array}$$

Written only in terms of the physical velocity, this becomes:

(21) $$\rho_{a}\frac{\partial v}{\partial t}+\rho_{a}v\cdot \nabla v=-\nabla p+\mu_{a}\nabla^{2}v-\varepsilon \frac{\mu_{a}}{k}v-\varepsilon^{2}\frac{c_{F}\rho_{a}}{k^{1/2}}v\left|v\right|$$

In the free-air domain, this equation becomes:

(22) $$\rho_{a}\frac{\partial u}{\partial t}+\rho_{a}u\cdot \nabla u=-\nabla p+\mu_{a}\nabla^{2}u$$

Here the porosity ε equals 38 %, and assumes that the fruit and the packaging are 'solid' materials through which no air can flow. The remaining volume is available for airflow.

##### Buoyancy

Note that buoyancy effects were not included in the model equations. Buoyancy implies gravity-driven airflow generated by temperature differences in the air, which in turn generates density differences. Neglecting buoyancy implies that no flow due to temperature-driven density differences is modeled. In this case, we assume that natural convection does not play a large role, so the airflow is largely pressure-driven, and only forced-convective airflow is accounted for. A key reason for this assumption is that the temperature does not impact the airflow field. As such, heat can be modeled as a passive scalar, and the airflow does not need to be recalculated each time step during the transient cooling process. This assumption greatly simplifies the solution procedure and reduces the computational cost. This is the reason why it is very rarely taken into account in CFD studies in postharvest engineering.

However, since airspeeds in the refrigerated container are rather low in the porous medium, we need to assess how important natural convective flow is in addition to forced convection. The validity of this assumption can be estimated based on the magnitude of buoyancy forces on the airflow by quantifying the Richardson number. The Richardson number (Ri) is the ratio of the buoyancy forces to the inertial forces:

(23) $$Ri=\frac{Gr}{{\text{Re}}^{2}}$$

The dimensionless Grashof (Gr) and Reynolds number (Re) are defined as:

(24) $$\begin{array}{c} Gr=\frac{\beta g\left(T_{fr,s}-T_{ref}\right){L_{ref}}^{3}}{\nu^{2}}\\ \text{Re}=\frac{U_{ref}L_{ref}}{\nu } \end{array}$$

Here, β is the thermal volumetric expansion coefficient [K^−1^], ν is the kinematic viscosity of air (1.461 × 10^−5^ m² s^−1^), g is the gravitational acceleration (9.81 m s^−2^), U_ref_ is a reference airspeed due to forced convective airflow which is in our case the physical airspeed in the porous medium [m s^−1^], L_ref_ is a characteristic length [m], for example, the diameter of the fruit or height of the stack of fruit. The stated temperature difference represents the difference between the regions of warm air, which induce a low air density, and the regions of cold air, which have a higher air density. In our case, this is the difference between the temperature of the fruit surface (T_fr,s_) and the local air temperature around the fruit (T_ref_ = T_a,loc_). Note that the Richardson number is defined here based on the local air temperature around the fruit, as these local air density differences will matter. The coefficient β can be taken as equal to 1/T for ideal gasses, so the average temperature of the air and the fruit surface. We can rewrite the Richardson number as:

(25) $$Ri=\frac{\frac{\beta g\left(T_{fr,s}-T_{ref}\right){L_{ref}}^{3}}{\nu^{2}}}{{\left(\frac{U_{ref}L_{ref}}{\nu }\right)}^{2}}=\frac{\beta g\left(T_{fr,s}-T_{a,loc}\right)L_{ref}}{{U_{ref}}^{2}}=\frac{g\left(T_{fr,s}-T_{a,loc}\right)L_{ref}}{\frac{\left(T_{fr,s}+T_{a,loc}\right)}{2}{U_{ref}}^{2}}=\frac{2gL_{ref}}{{U_{ref}}^{2}}\frac{\left(T_{fr,s}-T_{a,loc}\right)}{\left(T_{fr,s}+T_{a,loc}\right)}$$

We see that the Richardson number is inversely proportional to the square of the local airspeed and proportional to the temperature difference and the length scale. For Richardson numbers considerably smaller than one, the influence of buoyancy on the flow field can be neglected, and forced convective flow is found. In that case, heat is considered a passive scalar in the flow field, indicating that it does not influence the flow field significantly.

**Fig. 14:**
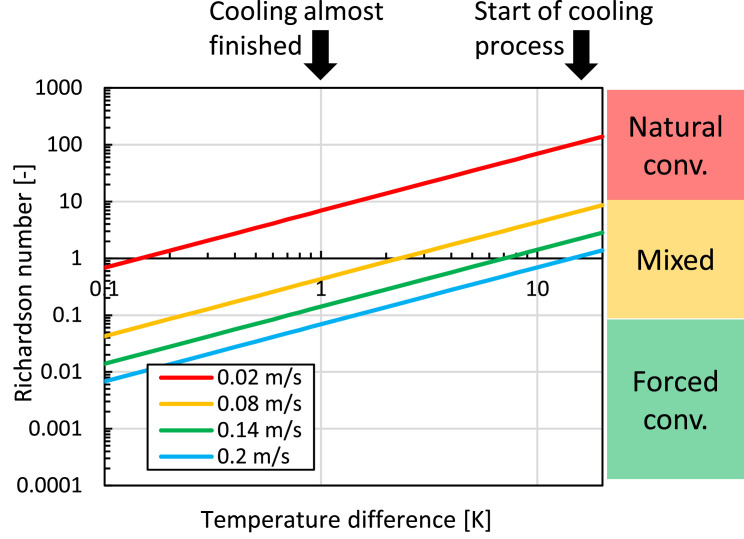
Richardson number as a function of the temperature difference between the fruit surface and the local air temperature for different airspeeds.

We use this analytical estimate to calculate the Richardson number as a function of the temperature difference between the local air temperature and the fruit surface temperature (Fig. 14). This temperature difference will change during the cooling process. We assume an airspeed in the porous stack of the fruit that lies roughly between roughly 0.02–0.2 m s^−1^ (U_ref_), depending on the location. The superficial airspeed in our study is 0.048 m s^−1,^ and the corresponding physical airspeed, for a porosity of 38 %, is 0.126 m s^−1^. The fruit diameter of 80 mm is taken as the length scale (L_ref_).

Based on the results and a chosen physical speed of 0.14 m s^−1^ (Fig. 14), the Richardson number implies that buoyancy forces are not negligible at the start of the cooling process in a refrigerated container. When the cargo is still warm at the start of the cooling process, they play an important role, or if the physical airspeeds get low. The surface temperature of the fruit rapidly drops within the first hour of cooling [64]. In most refrigerated containers, the temperature differences between warm and colder zones, such as fruit surface and local air temperature, are, however, rather limited. This analytical estimate suggests that for cargo that is not well ventilated (physical airspeed V <<<) due to a lot of airflow bypass, or cargo with high porosity (so V<<), and temperature differences above 1 K, buoyancy effects will play a role in addition to forced convection. Investigating the impact of buoyancy in such cases is a topic of further research, but will pose challenges to numerical stability, convergence, and computational cost.

##### Turbulence modeling

Airflow in the container and within the palletized cargo is turbulent. The Reynolds number at the inlet of the container is 36,300 when based on the inlet height (63.5 mm) and the airspeed (7.93 m s^−1^) entering the T-bar floor via the baffle plate. The Reynolds number within the porous medium is 730, based on a characteristic (physical) airspeed (0.126 m s^−1^) and a length scale that equals the diameter of the fruit (80 mm). Airflow will also be turbulent within the container since length scales are typically larger and airspeeds higher.

The continuity equation and Reynolds-averaged Navier-Stokes (RANS) equations for momentum and energy are solved using computational fluid dynamics (CFD). The airflow within the container and cargo is assumed to be a steady, turbulent, and incompressible fluid flow of dry air. These corresponding transport equations can be found in several fluid-mechanics textbooks. The conservation equation for momentum transfer (Eq. (17)) needs to be adjusted. In principle, only the dynamic viscosity of air μ_a_ is adjusted, so it equals the effective viscosity for turbulent flow (μ_a_ = μ_a,eff,T_ = μ_a,g_ + μ_T_). Here, μ_a,a_ is the molecular dynamic viscosity of air (1.79 × 10^−5^ kg m^−1^s^−1^), and μ_T_ is the turbulent viscosity [kg m^−1^s^−1^].

The Navier-Stokes equations for turbulent flow are solved by applying RANS with the standard k-ε turbulence model. This turbulence model is still the most commonly used in computational fluid dynamics (CFD) engineering and has been extensively tested [65]. This is an eddy-viscosity model, which assumes the isotropic behavior of turbulence. The model includes two additional turbulence transport equations for turbulent kinetic energy and turbulence dissipation. The turbulent viscosity (μ_T_), used to determine the Reynolds stresses, is calculated by assuming a specific relation between the transported variables, such as the turbulent kinetic energy (k_TM_ [m^2^ s^−2^]) and the turbulence dissipation rate (ε_TM_ [m^2^ s^−3^]). Here μ_T_ [kg m^−1^ s^−1^] equals:

(26) $$\mu_{T}=\rho C_{\mu }\frac{{k_{TM}}^{2}}{\varepsilon_{TM}}$$

Where Cμ is an empirical constant, often taken at 0.09. Note that eddy-viscosity models have some limitations:

- The turbulent viscosity is presumed to be an isotropic scalar quantity. This assumption implies that turbulence is isotropic as well. For many flow fields, this is not the case. The rapid expansion of the jet into the container enclosure in this study likely does not result in isotropic turbulence.
- The turbulent viscosity, used to calculate the Reynolds stresses, is determined by assuming a relation between the transported variables, namely the turbulent kinetic energy (k) and the turbulence dissipation rate (ε). This relation was established (empirically or theoretically) for specific flow conditions. No turbulence model is universally valid for all classes of flows [65]. As such, a model will perform better under specific flows than another.
- Most eddy viscosity models are developed to predict turbulence in the high-Reynolds number turbulent core of the flow. The model parameters are less accurate in the near-wall region, i.e., the low-Reynolds number region.

##### Challenges when modeling slot-ventilated enclosures

Refrigerated containers can be considered slot-ventilated enclosures. Air enters from the bottom of a refrigerated container at high speed into the enclosure and is extracted at the top. Much research has been done on this specific configuration [66], both on empty enclosures and enclosures filled with ventilated packaging, for example.

Modeling this flow field is rather challenging for several reasons. The airflow enters the cargo hold at high speed and then expands into a much larger space, by which a fast reduction in the speed occurs. The Coanda effect can make this air jet to remain attached to the bottom wall. This rapid airflow expansion combined with the Coanda effect is challenging to accurately model by RANS turbulence models. The jet can be laminar, turbulent, or in the transition regime. The Reynolds number in our refrigerated container is 35,000, based on the inlet speed at the slot inlet (7.9 m s^−1^) and the slot height (63.5 mm). At these Reynolds numbers, the jet is highly turbulent, so not in the transitional regime. In addition, the Coanda effect and flow expansion will also be affected by the fact that the container is filled with porous ventilated packaging and that the T-bar floor is present.

The main characteristics and findings of previous studies [66] are that often uniform inlet boundary conditions have been used, and most slots spanned the entire width of the enclosure. The Reynolds numbers of most studies were below 10,000, so slightly lower than in our refrigerated container. Clear differences between turbulence models have been found.

##### Boundary-layer modeling

Wall functions are used to model transport in the boundary layer. Wall functions are often preferred since they provide increased computational economy and easier grid generation compared to low-Reynolds number modeling (LRNM). Instead of resolving the boundary layer explicitly, these functions calculate the airflow in the boundary-layer region using semi-empirical functions. Despite being less accurate in some cases [67,68], wall functions are often the only viable option as LRNM grid generation and computation is challenging for complex 3-D configurations at high speeds [69,70]. In our model, very high airspeeds are found in some regions. Therefore it is challenging to mesh the boundary-layer region fine enough to apply low-Reynolds number modeling. Resolving the transport in the boundary layer on the no-slip surfaces (container walls, T-bar floor, etc.) required a huge computational cost. As such, wall functions are the most viable option in our case. In addition, our airflow field predictions are less prone to how the boundary layer is resolved compared to flow around aerodynamic bodies, for example. The reason is that airflow separation, for example, at the outlet or inlet, is prescribed by the geometry. For example, the edges of the T-bar floor are fixed separation points, so airflow separation is not impacted by transport processes in the boundary layer. In this case, the use of wall functions will not necessarily compromise accuracy. In our study, the dimensionless wall distance y^+^ was above 11 on the majority of the container walls. In these zones, the first grid cell lies outside the laminar sublayer or the buffer layer, meaning that the entire boundary layer is approximated by the wall functions. The highest values were reached near the inlet of the cargo hold, where y^+^ values up to 60 were found. As such, all computational cells lie within the lower part of the logarithmic layer of the boundary layer, where wall functions are valid.

##### Conservation equation for energy

The two-phase approach is used to solve heat transfer in the porous medium in the air and the fruit. This approach does not require thermal equilibrium between the air and the fruit. Fruit and air can have different temperatures. The fruit is, however, assumed to cool uniformly, by which core and surface temperatures are assumed equal. This approach is valid for low Biot numbers, where thermal gradients inside the fruit are not present. This implies sufficiently low airspeeds and/or small products to have a Biot number well below 1. For fruit stored in refrigerated containers, such low Biot numbers are found (Fig. 9). The conservation equations for a two-phase approach are in the fluid and solid domains:

(27) $$\begin{array}{c} \rho_{f}\frac{\partial \varepsilon_{fr}c_{p,f}T_{f}}{\partial t}+\rho_{f}u\cdot \nabla \varepsilon_{fr}c_{p,f}T_{f}=-\nabla \cdot \left(-\varepsilon_{fr}\lambda_{f}\nabla T_{f}\right)+A_{sf}h_{sf}\left(T_{s}-T_{f}\right)+S_{evap}\\ \rho_{s}\frac{\partial \left(1-\varepsilon_{fr}\right)c_{p,s}T_{s}}{\partial t}=-\nabla \cdot \left(-\left(1-\varepsilon_{fr}\right)\lambda_{s}\nabla T_{s}\right)+A_{sf}h_{sf}\left(T_{f}-T_{s}\right)+S_{resp} \end{array}$$

Here **u** is the superficial air velocity. When assuming a constant density, specific heat capacity, and porosity, and neglecting the source term for evaporation, we get the equation used in this study:

(28) $$\begin{array}{c} \rho_{f}c_{p,f}\frac{\partial T_{f}}{\partial t}+\rho_{f}c_{p,f}u\cdot \nabla T_{f}=-\nabla \cdot \left(-\lambda_{f}\nabla T_{f}\right)+\frac{A_{sf}h_{sf}}{\varepsilon_{fr}}\left(T_{s}-T_{f}\right)\\ \rho_{s}c_{p,s}\frac{\partial T_{s}}{\partial t}=-\nabla \cdot \left(-\lambda_{s}\nabla T_{s}\right)+\frac{A_{sf}h_{sf}}{\left(1-\varepsilon_{fr}\right)}\left(T_{f}-T_{s}\right)+\frac{S_{resp}}{\left(1-\varepsilon_{fr}\right)} \end{array}$$

The subscripts f and s indicate the fluid and solid phase, ρ is the density [kg m^−3^], c_p_ is the specific heat capacity [J kg^−1^K^−1^] (for air, 1005 J kg^−1^K^−1^ at 20°C), T is the temperature [K], λ is the thermal conductivity [W m^−1^K^−1^] (for air 0.0255 W m^−1^K^−1^ at 20°C). The heat exchange at the interface of the two materials is quantified by the convective heat transfer coefficient (h_sf_ [W m^−2^ K^−1^]) and the specific surface area for heat exchange between the fruit and the fluid A_sf_ [m^2^ m^−3^]. If required, additional source terms for respiratory heat generation or evaporative heat loss can be accounted for (S_resp_ [W m^−3^], S_evap_ [W m^−3^]). Often the contribution of respiration and evaporation is rather limited. Note that evaporation will cool down both the air around the fruit and the fruit surface. It was, however, now included as a source term in the air domain only. Other studies included this as a source term at the air-fruit interface as well. However, we neglected the evaporation source term in this study.

The specific surface area A_sf_ [m^2^ m^−3^], can be calculated for spherical fruit with radius R as:

(29) $$A_{sf}=\frac{A_{fr}}{V_{t}}=\frac{A_{fr}}{V_{fr}}\left(1-\varepsilon_{fr}\right)$$

(30) $$A_{sf}=\frac{4\pi R^{2}}{\frac{4}{3}\pi R^{3}}\left(1-\varepsilon_{fr}\right)=\frac{3}{R}\left(1-\varepsilon_{fr}\right)$$

Here, V_fr_ is the volume of fruit in the carton [m^3^], V_t_ is the total volume of the carton and A_fr_ is the surface area of the fruit [m^2^]. The porosity ε_fr_ of the fruit inside the cartons in the palletized fruit is calculated as:

(31) $$\varepsilon_{fr}=\frac{V_{a}}{V_{box,e}}=\frac{V_{box,e}-V_{fr,box}}{V_{box,e}}=1-\frac{V_{fr}N_{fr}}{V_{box,e}}$$

Here, N_fr_ is the number of fruit in a carton. The carton's volume is calculated using the external carton box dimensions (V_box,e_). The volume of the carton is neglected here for calculating the fruit porosity. Thermally, the carton will have little impact, which is why it is now considered thermally as air and not included in this porosity. In this study, the resulting fruit's porosity ε_fr_ is 47 %. The porosity of the carton, so accounting for both fruit and carton (ε), equals 38 % and is calculated as:

(32) $$\varepsilon =\frac{V_{a}}{V_{box,e}}=\frac{V_{a,i}}{V_{box,e}}=\frac{V_{box,i}-V_{fr,box,i}}{V_{box,e}}$$

Here V_a_ is the volume taken up by the air [m^3^]. Here we neglect the air space taken up by the ventilation openings (so V_a_ = V_a,i_). Note that this porosity is used in the momentum transport equation to calculate the actual physical speed that the fruit experiences around it.

In this study, the porosity ε_fr_ was used in the energy conservation equation (Eq. (28)), and ε was used in the momentum conservation equation (Eq. (20)). This porosity assumption ε_fr_ is essential to invoke a realistic thermal response since the carton would be considered fruit if a lower porosity (ε) were used. However, the porosity ε is essential to have the right physical speed inside the porous cargo since the carton leaves less space for the air to flow as it takes up a certain volume.

For turbulent flow, the energy conservation transport equation is adjusted. The conductive heat flux is defined as:

(33) $$q=-\lambda_{f,eff}\nabla T_{f}=-\left(\lambda_{f}+\lambda_{f,T}\right)\nabla T_{f}$$

Where the turbulent thermal conductivity of the air is defined as:

(34) $$\lambda_{f,T}=\frac{\mu_{T}c_{p,a}}{{\mathrm{Pr}}_{T}}$$

Here, Pr_T_ is the turbulent Prandtl number, μ_T_ is the turbulent viscosity, and c_p,a_ is the specific heat capacity of air. The turbulent Prandtl number is typically about 0.85. In our study, it was between 0.85 and 1.4 and was calculated in the software with a so-called Kays-Crawford approximation as:

(35) $${\mathrm{Pr}}_{T}={\left(\frac{1}{2{\mathrm{Pr}}_{T\infty }}+\frac{0.3c_{p,a}\mu_{T}}{\lambda_{a}\sqrt{{\mathrm{Pr}}_{T\infty }}}-{\left(\frac{0.3c_{p,a}\mu_{T}}{\lambda_{a}}\right)}^{2}\left(1-\exp\left(-\frac{\lambda_{a}}{0.3c_{p,a}\mu_{T}\sqrt{{\mathrm{Pr}}_{T\infty }}}\right)\right)\right)}^{-1}$$

Here, Pr_T∞_ is the Prandtl number at infinity and equals 0.85 and λ_a_ is the thermal conductivity of air.

##### Heat of respiration

Since the heat of respiration is quite low for oranges (at 5°C about 0.014–0.019 W kg^−1^ [46]), it is unlikely to have a significant impact on the cooling of fresh horticultural produce, as reported already for forced-air precooling [71,72]. Nevertheless, it was included in the model to make it generally valid for other fruit with high respiratory heat production. Examples are banana fruit or mangoes, where sensors can even pick up respiratory heat via an increase in temperature [73].

The heat of respiration can be included via a source term in the energy conservation equation for the heat of respiration in the fruit. The respiratory source term S_r_ is defined in the fruit domain as [W m_fr_^−3^].

(36) $$S_{r}=\rho_{fr}Q_{r}$$

**Fig. 15:**
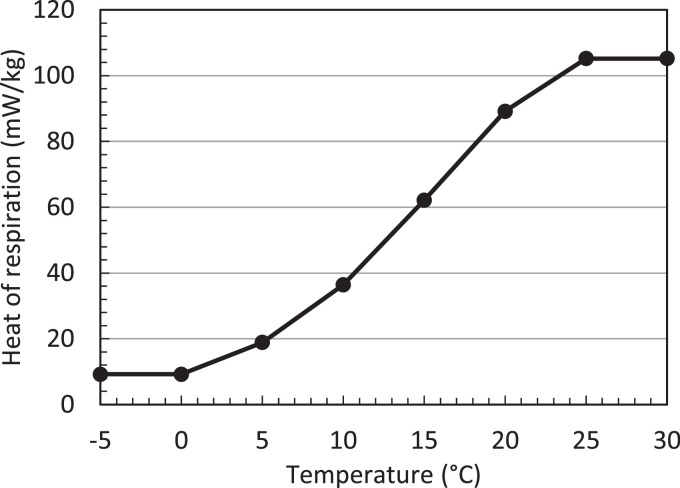
The heat of respiration from the literature by an interpolated function for orange fruit [46,74].

Here, the subscript *fr* indicates fruit, *ρ_fr_* is the density of the fruit [kg_fr_ m_fr_^−3^], and *Q_r_* is the mass-based heat of respiration [W kg_fr_^−1^]. Oranges have respiration heats that are temperature dependent and range from 9.2 mW kg_fr_^−1^ at 0°C up to 105 mW kg_fr_^−1^ at 25°C [46,74]. We took these literature data to determine respiration heat as a function of temperature by linear interpolation. A constant-value extrapolation was used for values above 25°C and below 0°C (Fig. 15). Note that these values are per cubic meter of citrus fruit. In the computational model, the volumetric source term is only accounted for in the fruit, not the entire porous medium. The entire cargo (26.3 tons) produces respiration heat of 2351 W at 20°C but only 498 W at 5 °C. The fruit is cooled down rapidly, so the respiration values are rather low.

##### Radiation exchange between the fruit

Long-wave radiation exchange between the fruit and the surrounding surfaces is not included. Long-wave radiation can be neglected inside the porous medium since the temperature difference between adjacent citrus fruit is small (typically < 1°C) during cooling, so limited radiation heat would be exchanged compared to convection heat transfer. The temperature differences between the pallets and the container walls are higher but also limited, and in these zones, the airspeeds are also high, so convective heat transfer will dominate.

##### Latent heat due to evaporation (transpiration) or condensation from and to the fruit

If the water evaporates from the fruit, heat is extracted from the fruit and the surrounding air, and additional air cooling occurs. The latent heat of evaporation is 2.5 MJ per kg of evaporated water. The heat extracted from the air by evaporation due to mass loss is small for refrigerated container transport of most fruit. The mass loss over a 3-week day trip is typically about 1-2 % [75,76]. Higher mass loss of about 7–10 % typically renders fruit unacceptable to the importer [77] but is common in shipments of flowers and plants. This amount implies 0.02 kg of evaporated water per kg of fruit. This amounts to a latent heat absorption of 28 mW kg^−1^ of fruit when averaged over a trip of 21 days. The evaporative heat loss is similar to the heat of respiration, which is low for oranges. The latent heat of evaporation resulting from the mass loss of the fruit was not included in the model. The condensation and evaporation that occurs in the refrigeration unit were not included, as the refrigeration unit of the container was not contained in the model.

##### Fruit quality loss

A kinetic rate law model was implemented to predict the evolution of fruit quality over time throughout the cold chain and its dependency on temperature. We do not target specific quality attributes, such as firmness, color, soluble solids content, color, vitamin C, flavor, or titratable acidity [78]. Our kinetic rate law model predicts the change of a general index of fruit quality [79]. This quality index quantifies the combined effect of the thermal history to which the fruit was subjected after harvest on the fruit quality. The quality index, therefore, accounts for thermal quality loss and not for transpiration or microbiologically-related quality decay. Next to this generic modeling of fruit quality and shelf life, individual quality attributes can be modeled using the same approach [73,80]. Below we present the modeling framework for a generic quality index and individual quality attributes. This study only targets modeling the thermally-driven general index of quality, which is later translated into the remaining shelf life (Section 1.6.2).

The kinetic rate law model quantifies the change of the index of fruit quality *A_i_* [81,82], but is also applicable to any other quality attribute:

(37) $$\frac{-dA_{i}}{dt}=k_{i}A_{i}^{n_{i}}$$

Here, the subscript *i* indicates the quality index or specific attribute (e.g., firmness), *k_i_* is the corresponding rate constant [s^−1^], and *n_i_* is the order of the reaction, which dictates if and how the rate is dependent on the value of A. The order of the reaction is dependent on the attribute's kinetics. Examples of zero-order reactions are browning due to the Maillard reaction, lipid oxidation, and enzymatic degradation [81,82]. A typical first-order reaction is vitamin loss. Many quality attributes, such as firmness and soluble solids content, actually depend on many biochemical reactions occurring in the fruit during maturation. The effect is lumped into a single attribute, serving as a measure of quality loss.

This ordinary differential equation (Eq. (37)) is solved. For a constant value of k, that is, at a constant temperature (as detailed below), the quality index or quality attribute decreases linearly over time (for zero-order reactions), where the magnitude of the slope equals k. An exponential decrease is found for first-order reactions at a constant temperature:

(38) $$A_{i}\left(t\right)=A_{0,i}-k_{i}\left(T\right)t$$

(39) $$A_{i}\left(t\right)=A_{0,i}e^{-k_{i}\left(T\right)t}+C_{i}$$

Here, A_0,i_ is the quality index or attribute at the start of the cooling process (t = 0 s), and C_i_ is an integration constant.

However, the rate constant k_i_ is not constant, and so Eq. (37) needs to be explicitly solved over time. The temperature dependency of the quality index or attribute was incorporated into the rate constant through an Arrhenius relationship (Robertson, 1993):

(40) $$k_{i}\left(T\right)=k_{0,i}e^{\frac{-E_{a,i}}{RT}}$$

Here, k_0,i_ is a constant [s^−1^], E_a,i_ is the activation energy [J mol^−1^], R is the ideal gas constant (8.314 J mol^−1^ K^−1^), and T is the absolute temperature [K]. The constants k_0,i_ and E_a,i_ were calibrated from quality index data as a function of time.

The exact procedure on how the parameters k_0_ and E_a_ were determined in this study for citrus fruit is explained and is based on [76]. The overall fruit quality index (*A_sl_*), linked to the remaining storage life (SL), was modeled using a first-order model. The following assumptions were applied based on information in the literature on 'Navel' orange fruit [83,84]. First, we assume orange fruit can be stored for approximately 70 d, so 10 weeks (*t_end_*) at 0°C. This implies *A_sl_ (0*°C, 0 h) = 100 % = *A_0,sl_* and *A_end,sl_*(0°C, t_end_ = 70 d so 10 weeks) = 20 %, so when the fruit is considered to be lost. This lower threshold can be chosen arbitrarily to some extent, as the choice does not affect the storage time at a certain temperature. The threshold affects the shape of the quality loss curve, however. Lower thresholds result in a curve that is more steeply decreasing. If data at different points in time is available for the fruit of interest at a certain temperature, a more precise choice of the threshold can be made. Second, we assume an increase in temperature of 10°C doubles the rate constant or decreases storage life by a factor of 2. This result means that the *Q_10_* value = 2 so that orange fruit can be stored for approximately 35 d at 10 °C. This *Q_10_* value equals the ratio of the rate constants at temperatures *T* and *T*+10K (Q_10_=*k_T+10_*/*k_T_*) and is typically about 2-3 for degradation reactions in fruit [81,85]. These values depend on the cultivar, harvest location, and exact ripeness classification. However, this study aimed to identify differences between treatments for orange fruit. As such, the conclusions will remain the same, even if slightly different values are taken for *t_end_* or Q_10_. Note that for different fruit species, the model can be recalibrated as the quality decay and the dependency on the temperature of the environment will be different. However, all fruits typically benefit from a hygrothermal environment with temperatures lower than the ambient temperature and relative humidity between 85 % and 95 %. Other technologies that change the gas composition of the air, such as controlled atmosphere storage, can also be used to prolong shelf life. In this case, the aforementioned quality models should also be recalibrated to account for that.

##### Calculation of parameters

Based on the relations above, these parameters, the constant k_0,i_, and activation energy *E_a,i_*, were calculated as follows.

(41) $$Q_{10}=\frac{k_{i,T+10}}{k_{i,T}}$$

(42) $$Q_{10}=\frac{k_{0,i}e^{\frac{-E_{a,i}}{R\left(T+10\right)}}}{k_{0,i}e^{\frac{-E_{a,i}}{RT}}}=\frac{e^{\frac{-E_{a,i}}{R\left(T+10\right)}}}{e^{\frac{-E_{a,i}}{RT}}}$$

Out of this *Q_10_* value equation, the activation energy is extracted, assuming *k_0,i_* is constant.

(43) $$\ln\left(Q_{10}\right)=\frac{-E_{a,i}}{R\left(T+10\right)}-\frac{-E_{a,i}}{RT}=\frac{-E_{a,i}T+E_{a,i}\left(T+10\right)}{RT\left(T+10\right)}=\frac{10E_{a,i}}{RT\left(T+10\right)}\approx \frac{10E_{a,i}}{R{\left(T+5\right)}^{2}}$$

(44) $$E_{a,i}\approx \frac{R{\left(T+5\right)}^{2}}{10}\ln\left(Q_{10}\right)=\frac{R{\left(T_{cal}+5\right)}^{2}}{10}\ln\left(Q_{10}\right)$$

This activation energy needed to be calibrated at a certain temperature *T_cal_*, as slight variation will occur and the value is not entirely constant. In this study, *T_cal_* = 273.15 K = 0 °C was chosen, so the temperature for which data was available. This leads to an exact match of the final quality attribute with the data at 0°C, but some discrepancy with the data at other temperatures. Once this activation energy is known, the constant *k_0,i_* is extracted:

(45) $$k_{0,i}=\frac{k_{i}\left(T_{cal}\right)}{e^{\frac{-E_{a,i}}{RT_{cal}}}}$$

The rate constant at a constant temperature (T_cal_), is calculated via Eq. (39), by known data on the remaining quality at this storage temperature (t_end_, A_i_(t_end_) = *A_end,sl_*) as:

(46) $$\begin{array}{c} A_{i}\left(t_{end}\right)=A_{0,i}e^{-k_{i}\left(T_{cal}\right)t_{end}}+C_{i}\\ k_{i}\left(T_{cal}\right)=-\frac{1}{t_{end}}\ln\left(\frac{A_{i}\left(t_{end}\right)-C_{i}}{A_{0,i}}\right) \end{array}$$

**Fig. 16:**
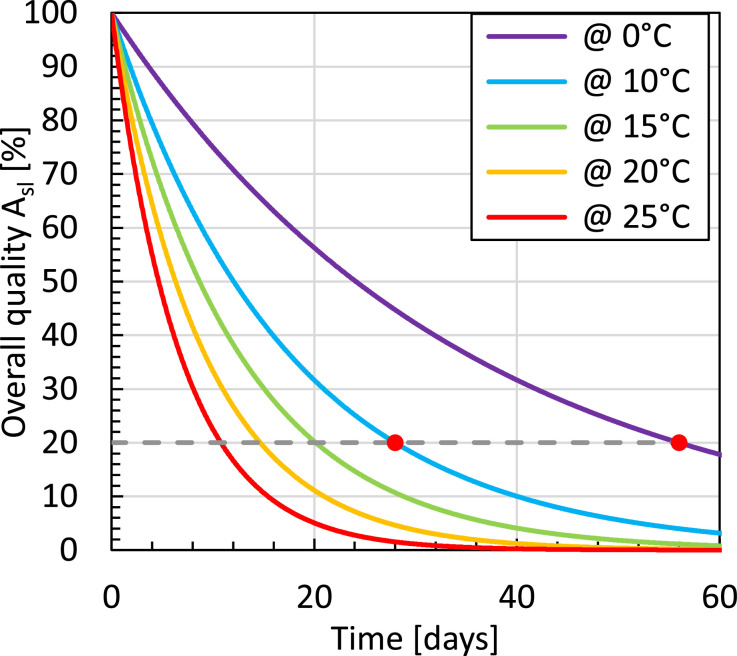
The overall quality index of orange fruit as a function of a constant storage temperature and time. The threshold value, where quality is considered to be fully lost (20 %), is shown by the dashed grey line. The red dots indicate the experimental calibration points where the quality index intersects with the threshold, namely at 70 d at 0°C and 35 d at 10 °C.

Using the data (t_end_ = 70 days, A_0,i_ = 1, A_i_(t_end_) = 0.2 and C_i_ = 0), we then get k_i_(T_cal_ = 0 °C) = 0.000958 h^−1^ or 2.66 × 10^−7^ s^−1^. This leads to *E_a,i_* = 44,585 J mol^−1^, *k_0,i_* = 321,947 h^−1^ or 89 s^−1^. We can use these data to calculate the quality index loss over time and the rate constant at any temperature. The model data at several temperatures are shown in Fig. 16 for the quality index. Note that the choice of A_end,sl,_ and T_cal_ affects the shape of the curve to some extent.

#### Constitutive equations

##### Convective heat transfer coefficient at the fruit surface

The convective heat exchange between the fruit and the airflow in the porous medium is quantified by a convective heat transfer coefficient (CHTC [W m^−2^ K^−1^]). This coefficient mainly depends on the fruit's size, shape, and airspeed approaching the fruit. We evaluate existing correlations for airflow in a packed bed of spherical particles and airflow around a single spherical fruit. We assume a single, spatially-constant CHTC over the entire fruit surface. A spatial CHTC variation can be present over the fruit surface [86,87], but this was not within the scope of the present study since we do not discretely model the single products. We highlight the correlations for flow around a single sphere and flow in a packed bed of spheres.

##### Single sphere

For a single sphere, we get [88]:

(47) $$Nu_{p}=2+\left(0.4{\text{Re}}_{p}^{0.5}+0.06{\text{Re}}_{p}^{0.667}\right){\mathrm{Pr}}^{0.4}{\left(\frac{\mu_{a}}{\mu_{a,wall}}\right)}^{0.25}$$

with ${\text{Re}}_{p}=\rho_{a}\frac{V.d_{p}}{\mu_{a}}=\frac{U.d_{p}}{\varepsilon \nu_{a}}$; $Nu_{p}=\frac{CHTC.d_{p}}{\lambda_{a}}$; $L_{ref}=d_{p};U_{ref}=V=\frac{U}{\varepsilon }$; $CHTC=\frac{Q}{A(T_{s}-T_{ref,loc})}=\frac{Q}{4\pi {\left(\frac{d_{p}}{2}\right)}^{2}\left(T_{s}-T_{ref,loc}\right)}=\frac{Q}{\pi {d_{p}}^{2}\left(T_{s}-T_{ref,loc}\right)}$

Here, μ_a_ and μ_a,wall_ are the absolute viscosities of the air (subscript a) and the air at the wall, respectively, which were considered as equal in this study (μ_a_ = 1.79 × 10^−5^ kg m^−1^ s^−1^) as the air properties were assumed constant; Pr is the Prandtl number (Pr = ν_a_/α_a_), which was 0.7 in the present study. Here, ν_a_ is the kinematic viscosity of air [m^2^ s^−1^], which equals μ_a_/ρ_a_ and T_ref,loc_ is the local approach flow temperature of the air that impinges on the sphere. Q is the heat flow rate [W] and A is the sphere's surface [m^2^]. In this single-sphere correlation, the airspeed is the physical airspeed (V) since this is the airspeed around the sphere. Using these equations, the CHTC can directly be calculated as:

(48) $$\begin{array}{c} Nu_{p}=\frac{CHTC.d_{p}}{\lambda_{a}}=2+\left(0.4{\text{Re}}_{p}^{0.5}+0.06{\text{Re}}_{p}^{0.667}\right){\mathrm{Pr}}^{0.4}{\left(\frac{\mu_{a}}{\mu_{a,wall}}\right)}^{0.25}\\ =2+\left(0.4{\left(\frac{U.d_{p}}{\varepsilon \nu_{a}}\right)}^{0.5}+0.06{\left(\frac{U.d_{p}}{\varepsilon \nu_{a}}\right)}^{0.667}\right){\mathrm{Pr}}^{0.4}{\left(\frac{\mu_{a}}{\mu_{a,wall}}\right)}^{0.25} \end{array}$$

(49) $$CHTC=\frac{\lambda_{a}}{d_{p}}\left(2+\left(0.4{\left(\frac{U.d_{p}}{\varepsilon \nu_{a}}\right)}^{0.5}+0.06{\left(\frac{U.d_{p}}{\varepsilon \nu_{a}}\right)}^{0.667}\right){\mathrm{Pr}}^{0.4}{\left(\frac{\mu_{a}}{\mu_{a,wall}}\right)}^{0.25}\right)$$

This equation lets us calculate the CHTC directly from the superficial airspeed, porosity, particle diameter, and air properties. Note that the porosity here assumes that the fruit and the packaging obstruct airflow and are considered solid materials.

##### Packed bed

For a packed bed, we get [88]:

(50) $$Nu_{pb}=\left(0.5{{\text{Re}}_{pb}}^{0.5}+0.2{{\text{Re}}_{pb}}^{0.667}\right){\mathrm{Pr}}^{0.33}22<Re_{pb}<8,000$$

with

$$Nu_{pb}=\frac{CHTC.L_{ref,pb}}{\lambda_{a}}=\frac{CHTC.d_{p}}{\lambda_{a}}\frac{\varepsilon }{1-\varepsilon };Nu_{pb}=\frac{\varepsilon }{1-\varepsilon }Nu_{p}$$

$${\text{Re}}_{pb}=\frac{V.L_{ref,pb}}{\nu_{a}}=\frac{\frac{U}{\varepsilon }.d_{p}\frac{\varepsilon }{1-\varepsilon }}{\nu_{a}}=\frac{U.d_{p}}{\nu_{a}\left(1-\varepsilon \right)};{\text{Re}}_{pb}=\frac{\varepsilon }{1-\varepsilon }{\text{Re}}_{p}$$

$$L_{ref,pb}=d_{p}\frac{\varepsilon }{1-\varepsilon };U_{ref}=V=\frac{U}{\varepsilon },d_{p}=\frac{6V_{p}}{A_{p}}=\frac{6\frac{4}{3}\pi {r_{p}}^{3}}{4\pi {r_{p}}^{2}}=\frac{6r_{p}}{3}=2r_{p}$$

$$\begin{array}{c} CHTC=\frac{Q}{A_{s}\left(T_{s}-T_{ref,af}\right)}\\ CHTC=\frac{Nu_{pb}\lambda_{a}}{d_{p}}\frac{1-\varepsilon }{\varepsilon } \end{array}$$

Here T_ref,af_ is the approach flow temperature of the air that enters the packed bed. As the energy conservation equation in the physics-based model relies on the local fluid temperature in the packed bed (Section 1.3.2.2), this correlation is likely less accurate since it was derived for an entire packed bed using the approach flow temperature. Using these equations, the CHTC can directly be calculated as:

(51) $$Nu_{pb}=\frac{CHTC.d_{p}}{\lambda_{a}}\frac{\varepsilon }{1-\varepsilon }=\left(0.5{{\text{Re}}_{pb}}^{0.5}+0.2{{\text{Re}}_{pb}}^{0.667}\right){\mathrm{Pr}}^{0.33}=\left(0.5{\left(\frac{\frac{U}{\varepsilon }.d_{p}\frac{\varepsilon }{1-\varepsilon }}{\nu_{a}}\right)}^{0.5}+0.2{\left(\frac{\frac{U}{\varepsilon }.d_{p}\frac{\varepsilon }{1-\varepsilon }}{\nu_{a}}\right)}^{0.667}\right){\mathrm{Pr}}^{0.33}$$

(52) $$CHTC=\frac{\lambda_{a}}{d_{p}}\frac{1-\varepsilon }{\varepsilon }\left(0.5{\left(\frac{\frac{U}{\varepsilon }.d_{p}\frac{\varepsilon }{1-\varepsilon }}{\nu_{a}}\right)}^{0.5}+0.2{\left(\frac{\frac{U}{\varepsilon }.d_{p}\frac{\varepsilon }{1-\varepsilon }}{\nu_{a}}\right)}^{0.667}\right){\mathrm{Pr}}^{0.33}$$

This equation enables us to calculate the CHTC directly from the superficial airspeed, porosity, particle diameter, and air properties.

Note that these empirical correlations were derived for forced convection. Buoyancy effects were not explicitly accounted for, as these depend on the temperature difference between the air and the fruit. The packed bed correlation was derived for a Re_pb_ range from 22 to 8000. The single sphere correlation is valid for a Re_p_ range from 3.5 to 76,000. These Reynolds number ranges for both correlations cover the airspeeds in packed fruit in refrigerated containers.

**Fig. 17:**
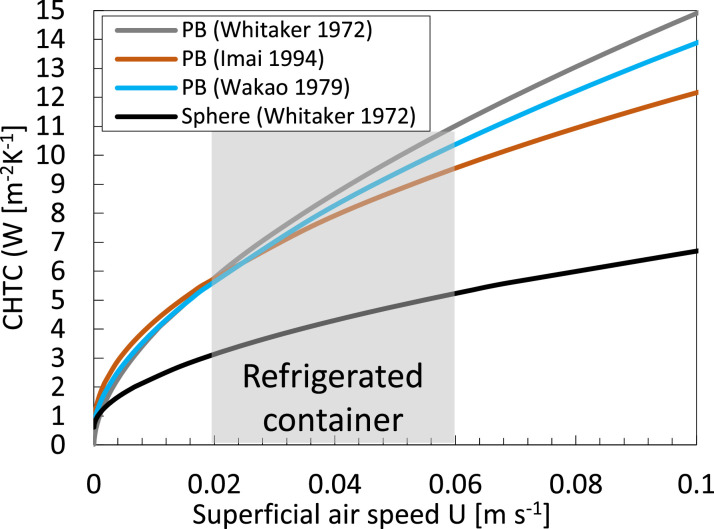
CHTC as a function of the superficial airspeed in a packed bed of spherical fruit and for a single spherical fruit of 80 mm with a porosity of 38 %. The superficial airspeed range used for refrigerated containers is indicated with the grey area.

These CHTC correlations are shown as a function of the superficial airspeed in Fig. 17. Also, other correlations for packed beds are shown here. Note that these packed bed correlations were originally defined as functions of Nu_pb_' and Re_p_^'^. Re_p_^'^ is defined here based on the superficial airspeed and not physical airspeed, as used above. All these correlations are independent of the porosity as they include the superficial airspe on a length scale equal to the particle diameter.

(53) $$Nu{{}^{\prime }}_{pb}=2+1.1\text{Re}{{{}^{\prime }}_{p}}^{0.6}{\mathrm{Pr}}^{0.33}$$

15 < Re_p_' < 8500 [43,55] with $N{u_{pb}}^{\prime }=\frac{CHTC.d_{p}}{\lambda_{a}}$; $\text{Re}{{}^{\prime }}_{p}=\rho_{a}\frac{V\varepsilon .d_{p}}{\mu_{a}}=\frac{U.d_{p}}{\nu_{a}}$

(54) $$Nu{{}^{\prime }}_{pb}=2+1.8{{\text{Re}}_{p}}^{0.5}{\mathrm{Pr}}^{0.33}$$

50 < Re_p_' < 5000 [89] with $Nu{{}^{\prime }}_{pb}=\frac{CHTC.d_{p}}{\lambda_{a}}$; $\text{Re}{{}^{\prime }}_{p}=\frac{U.d_{p}}{\nu_{a}}$

We conclude that there is a clear difference between the single-sphere CHTC and the CHTC predicted for packed beds. The packed bed correlations all agree well with one another. In the superficial airspeed range of a refrigerated container, the CHTCs for a single sphere and a packed bed vary between about 3-5 W m^−2^K^−1^, and 5–11 W m^−2^K^−1^, respectively. In this study, we used the correlation for a single sphere (Eq. (49)) since this gives us the local CHTC of every single particle as a function of the local superficial speed.

##### Different definitions for single sphere and packed bed

For a single sphere, Re_p_ is the particle Reynolds number (Eq. (11)) in these equations. This Reynolds number (Re_p_) was based on the air speed of the approach flow (U_ref_), so the physical airspeed (V) and length scale of the fruit (L_ref_). This length scale for spherically-shaped products is the (equivalent) diameter of the sphere (d_p_), as this is representative of the flow structures behind the sphere (e.g., vortices), making it equivalent to the particle Reynolds number. Nu_p_ is the particle Nusselt number and equals CHTC.L_ref_/λ_a_, where λ_a_ is the thermal conductivity of air (0.0242 W m^−1^ K^−1^) and the length scale equals d_p_.

(55) $${\text{Re}}_{p}=\rho_{a}\frac{V.d_{p}}{\mu_{a}}=\frac{U.d_{p}}{\varepsilon \nu_{a}}$$

For a packed bed, Re_pb_ is the particle Reynolds number in the packed bed. This Reynolds number was sometimes defined in a different way, namely where the characteristic length scale is a measure of the size of the void spaces [88]. Both Reynolds and Nusselt numbers are a function of the porosity which implies that the impact of the void space and packing density on the airflow and corresponding heat transfer is included in the correlations. The correlation can be rewritten as:

(56) $$\begin{array}{c} Nu_{pb}=\left(0.5{{\text{Re}}_{pb}}^{0.5}+0.2{{\text{Re}}_{pb}}^{0.667}\right){\mathrm{Pr}}^{0.33}\\ Nu_{p}=\left(0.5{\left(\frac{\varepsilon }{1-\varepsilon }{\text{Re}}_{p}\right)}^{0.5}+0.2{\left(\frac{\varepsilon }{1-\varepsilon }{\text{Re}}_{p}\right)}^{0.667}\right){\mathrm{Pr}}^{0.33}\frac{1-\varepsilon }{\varepsilon } \end{array}$$

Note that both of these correlations were defined using different Reynolds and Nusselt numbers. The difference is the length scale used, where both use the local (physical) airspeed. This should be accounted for when extracting the convective heat transfer coefficients from one of these correlations or comparing them.

#### Material properties

The applied material properties are given in Table 2 and their sources. The fruit shape, size, and material properties were assumed to be identical for all cases. Note that all these can differ according to the harvest time and place and due to the biological variability between individual fruit in a batch. This variability comes from variations in the local growing conditions of each tree and within each tree, ripeness degree, harvest location, and harvest year. Small differences in thermal properties between different literature sources are present [90]. However, the rationale of the present study was to target specific questions about the cooling and quality evolution, and our simplified configuration is sufficient to serve this purpose. The material properties of fruit were taken constant, thus independent of temperature, which was sufficient for the scope of this study. The thermal properties of air were slightly dependent on temperature but varied little in the range of temperatures considered in the study. Note that the carton cardboard was neglected in the thermal properties of the porous medium.

**Table 2:** Thermal properties of the materials.

	Density [kg m^−3^]	Specific heat capacity [J kg^−1^K^−1^]	Thermal conductivity [W m^−1^K^−1^]	Dynamic viscosity [kg m^−1^ s^−1^]	Source
Fruit	955	3598	0.543	-	[76]
Air (at 0°C & 20°C)	1.29 - 1.21	1004.8 - 1005.4	0.0242 - 0.0258	1.71×10^−5^ - 1.81×10^−5^	[91]

#### Porous-medium parameters & calibration

We determine and calibrate the porous medium parameters for the palletized fruit. We model flow through 80 cartons of 80 mm orange fruit, with a porosity of 38 % (including the fruit and the cardboard boxes for airflow) via the porous media approach. The porous-medium parameters in the volumetric sink term in the Navier-Stokes equations for Darcy and Forchheimer are determined via a calibration procedure. In this study, we did not account for the Brinkman term. For a refrigerated container, this term accounts for the viscous losses at the container walls. They are expected to have a limited impact.

The first step is to obtain empirical data on the pressure loss (Δp [Pa]) versus flow rate (G_a_ [m^3^ s^−1^]) through the entity that we want to model. In our case, this entity is a pallet of fruit. These empirical data can come from wind tunnel experiments [92] and CFD simulations [2] on a similar geometrical setup using the same fruit and packaging. The flow rate G_a_ equals A.U, where A [m^2^] is the surface area perpendicular to the flow direction, and U is the superficial airspeed entering the porous medium.

**Fig. 18:**
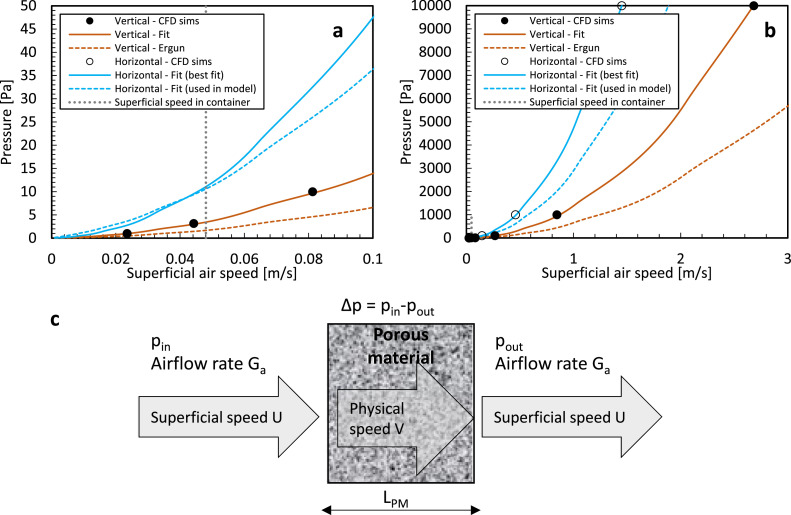
Pressure loss over a pallet in the vertical and horizontal direction as a function of the flow rate through the pallet, as well as the results from the Ergun equations and vertical flow through a stack of spheres without packaging. The data points from the CFD simulations (dots), as well as the second-order polynomial approximations, are shown. (a) zoomed in on the speed range relevant for refrigerated containers, (b) full range for fitting, (c) schematic of the speed in a porous medium.

Second, the volumetric sink term in the momentum equation (Eq. (17)) is rewritten to estimate this source term in the Navier-Stokes equations based on the empirical data. We rewrite it in terms of the pressure drop over the porous medium Δp [Pa], the average superficial airspeed in the porous medium U and the path length that the flow needs to travel in the porous medium L_PM_, but not accounting for tortuosity (Fig. 18). When also substituting the average superficial airspeed into the sink term, we get from Eq. (8), when neglecting the Brinkmann term:

(57) $$\begin{array}{c} S_{m,PM}=\nabla p=-\frac{\Delta p}{L_{PM}}=-\frac{\mu_{a}}{k}u-\frac{c_{F}\rho_{a}}{k^{1/2}}u\left|u\right|=-\frac{\mu_{a}}{k}U-\frac{c_{F}\rho_{a}}{k^{1/2}}U^{2}\\ \Delta p=\frac{\mu_{a}}{k}L_{PM}U+\frac{c_{F}\rho_{a}}{k^{1/2}}L_{PM}U^{2} \end{array}$$

The second term in the equation can be rewritten into the well-known form with the drag coefficient C_D_, where C_D_ [m^−1^] or C_D_'' [kg m^−4^] equals 2 c_F_/k^1/2^:

(58) $$\begin{array}{c} \Delta p=\frac{c_{F}\rho_{a}}{k^{1/2}}U^{2}L_{PM}=\frac{1}{2}\frac{2c_{F}}{k^{1/2}}\rho_{a}U^{2}L_{PM}=\frac{1}{2}C_{D}\rho_{a}U^{2}L_{PM}=\frac{1}{2}{C_{D}}^{\prime \prime }U^{2}L_{PM}\\ C_{D}=\frac{2c_{F}}{k^{1/2}}\\ {C_{D}}^{\prime \prime }=\frac{2c_{F}}{k^{1/2}}\rho_{a} \end{array}$$

We fit the empirically obtained data points ((Δp_1_, U_1_), (Δp_2_, U_2_), (Δp_3_, U_3_), …) with Eq. (57) of a second-order polynomial to obtain the polynomial parameters a and b. From these two parameters, we can inversely determine the coefficients c_F_ and k that provide the best fit to the model:

(59) $$\begin{array}{c} \Delta p=\frac{\mu_{a}}{k}L_{PM}U+\frac{c_{F}\rho_{a}}{k^{1/2}}L_{PM}U^{2}=aU+bU^{2}\\ a=\frac{\mu_{a}}{k}L_{PM};b=\frac{c_{F}\rho_{a}}{k^{1/2}}L_{PM}\\ k=\frac{\mu_{a}}{a}L_{PM};c_{F}=\frac{bk^{1/2}}{\rho_{a}L_{PM}}=\frac{b{\left(\frac{\mu_{a}}{a}L_{PM}\right)}^{1/2}}{\rho_{a}L_{PM}} \end{array}$$

In this study, the porous-medium parameters are determined for a pallet of Supervent packaging from previous CFD studies for vertical and horizontal flow through the pallet. For vertical flow, the pallet was simplified as a single stack of cartons [36]. For horizontal flow, the pallet was simplified as a single layer of cartons [1]. We assumed here that the resistance in both horizontal directions is the same. The differences between both directions are relatively small compared to those with the vertical flow.

These empirical data and the best model fits are shown in Fig. 18. Also, the results from the Ergun equations (Eq. (14)) for a stack of spherical products are given. The porous medium coefficients for the Darcy and Forchheimer terms are given in Table 3 in the vertical and horizontal directions. Note that the pressure-velocity data were fitted with a regression on the points at the lowest airspeeds (vertical: 0.02354 – 0.265 m s^−1^, horizontal: 0.144-0.457 m s^−1^). The reason is that the fit becomes inaccurate at low airspeeds when we also include fitting the data points at higher speeds. These coefficients and implementation were validated on a 2D model of a single vertical stack of cartons (Section 2.1).

**Table 3:** Porous medium coefficients derived from fitting data.

Configuration	Direction	Darcy	Forchheimer	C_D_ [m^−1^]	Refs.
		k [m^2^]	c_F_ [-]		
Palletized orange fruit	Vertical	3.750×10^−6^	1.014	1048	[36]
Palletized orange fruit	Horizontal (used in model)	1.92×10^−7^	1.014	4630	[1]
Palletized orange fruit	Horizontal (best fit with data)	1.36×10^−6^	4.53	7774	[1]

We find that for a pallet of fruit, the airflow resistance in the horizontal direction is higher than in the vertical direction. This means that horizontal flow will be more suppressed than vertical flow, so air prefers to travel vertically through the pallets. This corresponds to the much lower total open area of the vent holes (3 %) compared to the vertical direction (13 %). The airflow resistance for a stack of spheres without cartons, calculated via the Ergun equations, is lower than with cartons. As such, the Ergun equations are not representative of estimating pressure losses over palletized fruit packed in ventilated packaging, which is in accordance with previous findings [93,13]. Also for this packaging type, the ventilated packaging seems to be a key contributor to the pressure resistance over the pallet.

From the ratio of Forchheimer to Darcy terms for a refrigerated container (Fig. 12), both terms are relevant to include for the present pallet of Supervent. The pressure resistance in the vertical and horizontal direction of the pallet was found to be different. In this study, the porous medium was modeled anisotropically for the Darcy and the Forchheimer terms. However, it was not possible to implement an anisotropic value of the Forchheimer coefficient (c_F_) in the software used. Therefore, we used the same c_F_ in both directions, namely the one for the vertical flow. We then refitted the value of the horizontal Darcy permeability (k), so it agrees as well as possible with the horizontal fit (Table 3, ‘Horizontal (best fit with data)’) in the lower speed range. We get a slightly different permeability (Table 3, ‘Horizontal (used in model)’), but the data agree well in the speed range in a refrigerated container (∼ 0.05 m s^−1^). These data (Table 3, ‘Horizontal (used in model)’) were implemented in the model for the anisotropic porous medium. The discrepancies of this fit will be higher at speeds that are higher than those found in a refrigerated container.

Since the airflow rate is prescribed in the simulations, the airflow resistance will affect only the flow field and pressure distribution inside the container, not the airflow rate through the refrigerated container. In reality, the higher pressure resistance of the cargo hold will lead to a different operating point for the evaporator fans. However, given the low-pressure loss over the cargo hold (∼10^1^ Pa), compared to the pressure delivered by the fans (∼10^2^ Pa), the impact of the cargo on the operating point is expected to be rather low.

#### Boundary and initial conditions

The boundary conditions for airflow and heat transfer in the container are depicted in Fig. 13. The boundary conditions for the base case are detailed. Other variants that were simulated are discussed elsewhere. Airflow was solved in a steady state since the airflow rate is kept constant over time. We assume that the fruit was at ambient temperatures after harvest for solving heat transfer. The fruit is cooled down within the container. This case is representative of the ambient or 'warm' loading of refrigerated containers [75,22]. This practice alleviates the limited throughput of precooling facilities during the peak harvest season by using the containers as mobile precoolers. The initial temperature of the fruit and cardboard boxes was equal to the characteristic fruit temperature after being packed and palletized, typically between 10 and 20°C for South Africa. In this study, we assumed an initial temperature of 16°C.

At the inlet of the domain, a constant airflow rate is imposed. A uniform airspeed is imposed. Its magnitude is based on the flow rate of evaporator fans in the refrigeration unit of the container. We evaluated the high-speed flow rate (4150 m^3^ h^−1^, Table 1), which induced a speed of 7.9 m s^−1^ at the inlet slot of the cargo hold. The corresponding specific flow rate, i.e., per fruit mass, is 0.0437 L s^−1^kg^−1^. A constant air temperature was imposed at the inlet for the base case, the so-called delivery-air temperature (DAT). This assumption implies that the refrigeration unit can cool the return air entirely back to the set temperature, which is often the case in practice. The measured delivery air temperature from sensor data was also used in other simulations, such as the validation simulations, where the set delivery air temperature was set at 1°C. The inlet (DAT) air temperature was 2°C. The reason for this low temperature is that this research was conducted for the South African citrus industry, which exported 2.4 million tons during the 2021 season, making it the second-largest exporter of fresh citrus worldwide [94]. Some export markets require a cold disinfestation treatment as a risk mitigation protocol for pests associated with the fruit export pathway. This protocol entails a lower throughput in the precooling facilities, and this temperature must be maintained during overseas transport. Ambient loading is particularly of interest to the South African citrus industry. Since the container has sufficient refrigeration capacity to cool the fruit within 3-6 days [95,96,22], the use of ambient loading significantly reduces demand for precooling facilities, which are currently in short supply.

At the outlet, the ambient atmospheric pressure was imposed, and a thermal outflow condition, which assumes no thermal gradients in the flow direction. The container walls were modeled as no-slip walls with zero roughness. The container walls on the top, bottom, and door end exchanged heat with the environment. The heat flux q [W m^−2^] was calculated as:

(60) $$q=U_{2D,eq}\left(T_{ext}-T_{s}\right)$$

Where U_2D,eq_ is the thermal transmittance (0.71 W m^−2^ K^−1^, (Section 1.3.1)), T_ext_ is the external air temperature, which was taken equal to 20°C. Note that for heat load calculations, often higher temperatures are used, such as 38°C [46,22], and T_s_ is the surface temperature of the interior container wall, which results from the simulation.

At the interface between the porous medium (pallets) and the air, also internal boundary conditions needed to be specified for heat transfer and airflow. For airflow, the continuity of fluid flow and temperature was modeled.

The vertical wall at the refrigeration unit end is modeled as an internal, thin stainless steel wall (1 mm). Heat can be transferred by conduction from the refrigeration unit end to the cargo hold. Due to the high conductivity of the wall, the temperature on both sides will be quite similar. The T-bar floor is also modeled as an internal, thin aluminum wall (1 mm). The void plug blockage was modeled as a 1 mm thick plastic coated paper material.

#### Time scales

We include a discussion on the different time scales for cooling and food quality decay. More details can be found in [73]. The reason is that we typically observe that the temperature differences among different speeds or within the fruit are substantial. Yet, their impact on fruit quality attributes can appear relatively insignificant. This phenomenon can be elucidated by analyzing the time constants associated with cooling and quality decay. For an exponential function, the time constant of parameter X is defined as follows.

(61) $$X\left(t\right)=X_{0}e^{-t/\tau }$$

In the proposed first-order reaction kinetics model for overall quality, as well as some individual quality attributes described by a similar exponential function, the time constant can be derived as τ = 1/k_i_(T). Furthermore, the temperature-time evolution during the cooling process can be represented by the following exponential function [97,98], where P denotes the cooling coefficient.

(62) $$Y\left(t\right)=e^{-Pt}$$

Hence, the time constant can be determined as τ = 1/P. The time constants for overall quality decay, various quality attributes, and the cooling process (at each speed) were found to be substantially lower - typically more than three orders of magnitude - than those pertaining to quality decay, indicating a significantly faster cooling process [73]. Notably, the time constants for cooling are highest at low speeds, making them more akin to those of quality decay. Consequently, in typical forced convective cooling scenarios, the process often occurs rapidly, thereby limiting the impact of cooling rate differences on quality decay. However, in forced convective cooling within ventilated palletized packaging, both high speeds near vent holes and low speeds due to dead zones can be encountered. As a result, within a cargo, time constants for cooling can exhibit significant variations in practice, even within a single unit operation.

The time constant for fruit quality decreases as the fruit ripens, underscoring the imperative of faster and more uniform cooling for ripe fruit. For the quality attributes, all of them demonstrate equal susceptibility to temperature, exhibiting similar time constants. In conclusion, employing high airflow rates during post-harvest cooling is crucial, particularly for ripe fruit. However, the variations in quality within the fruit remain relatively constant and independent of the air-cooling speed. Note that the time constant for turbulent airflow is much lower than that of cooling.

### Spatial and temporal discretization

**Fig. 19:**
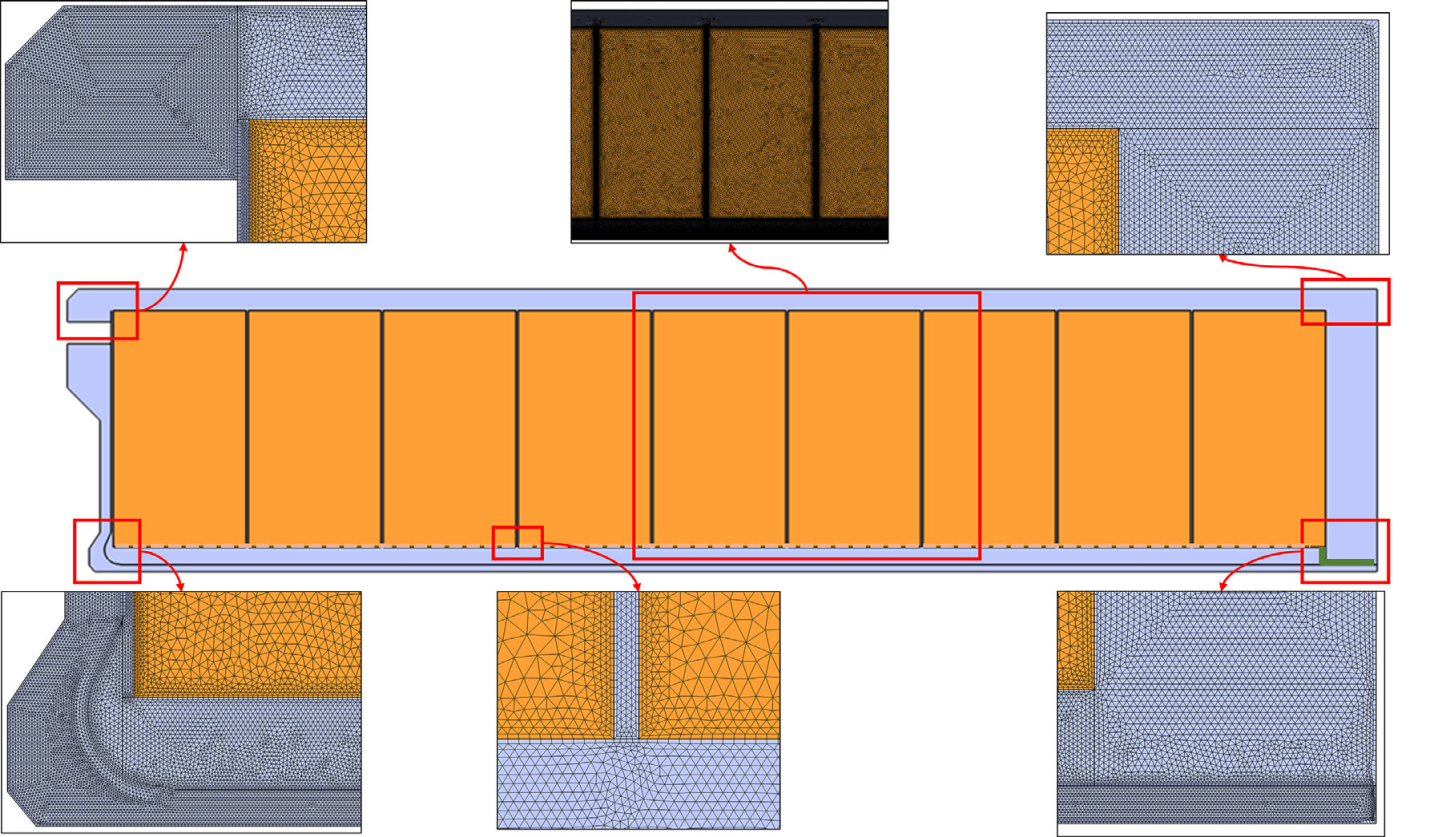
Computational grid that was used in the simulations, with several details highlighted (pallets with fruits are depicted in orange).

An appropriate grid (Fig. 19) was constructed for the 2D container based on a grid sensitivity analysis. The grid consists of 487,803 quadrilateral and triangular finite elements. A few layers of quadrilateral elements were used on the walls, and then a transition to triangular elements was implemented. A gradual refinement toward the air–porous medium and air-container wall interfaces was applied to enhance numerical accuracy and stability, as the largest gradients occur there, including at the start of the cooling process. Also, the grid at the inlet and bottom cavity (T-bar floor and pallet base) was refined since high airspeeds occur at these points. The transient simulations applied adaptive time-stepping, with a maximal time step of 600 s. This time step was determined from sensitivity analysis and ensured a sufficient temporal resolution for the output data. Note that refrigerated containers typically log temperature data at a 1 h interval, and sensors used in the cold chain during precooling or refrigerated transport log at a 10–15 min interval.

### Numerical implementation and simulation

This model was implemented in COMSOL Multiphysics (version 6.1). COMSOL is a finite element-based commercial software. Airflow in the air and inside the porous medium (containing air and fruit) was solved using the 'Free and Porous Media Flow' physics interface. The steady-state airflow was solved only once before the transient heat transfer calculation. Since the flow field was assumed steady and stable over time, as no buoyancy was included in the model, the flow field did not need to be recomputed anymore during the transient simulations. Thus the flow equations were switched off during transient calculations. Transient heat transfer in the air and the fruit during convective air cooling was solved using the ‘Heat Transfer in Solids and Fluids' interface. Heat transfer in the fruit and the air were coupled using the 'Nonisothermal Flow' interface. The interfacial conditions between fruit and air were defined via the convective heat transfer coefficient. The kinetic rate law model for the fruit quality was implemented using the ‘Ordinary Differential Equation’ interface. The equations were solved for the dependent variables velocity **u** (u,v,w), air temperature T_a_, fruit temperature T_fr_, and fruit quality index A. The quality attributes do not affect temperature and could be solved separately after the thermal calculations. We used the following discretization schemes: P2+P2 for airflow and quadratic Lagrange elements for heat transport in the fruit and the air. A segregated direct solver was used for steady-state airflow, relying on the GMRES solver for fluid flow and the PARDISO solver for turbulence. A fully-coupled direct solver was used for transient heat transfer airflow, relying on the MUMPS (MUltifrontal Massively Parallel sparse direct Solver) solver scheme. The tolerances for convergence and other solver settings were determined based on sensitivity analysis, so further increases in the tolerance did not further alter the solution results. These tolerances were 10^−4^ for the steady-state calculation and 10^−4^ for the transient simulation.

Before simulating the transient cooling process, steady-state simulations were performed to obtain the flow field and the initial temperature conditions. During these simulations, the temperature of the fruit was fixed to its initial value (16°C), and the inlet temperature was taken equal to the set point of the container air temperature (2°C). After the steady-state simulations, transient simulations of the precooling process were performed. During the transient simulations, the airflow field did not need to be recomputed anymore. The computational cost was significantly reduced since only the energy equation needed to be solved over time. The two-step approach is often applied for forced-air cooling applications.

### Metrics to evaluate cooling & fruit quality

#### Seven-eighths cooling time

The output of the simulations is the history of the average fruit temperature at each point in the container. For the cooling kinetics, the cooling rate of each fruit was assessed from the fruit temperature profiles. For this, the fractional unaccomplished temperature change (*Y*) was determined:

(63) $$Y=\frac{T_{fr}\left(t\right)-T_{DAT}}{T_{fr,0}-T_{\;DAT}}$$

Here, subscripts *fr,0* and *DAT* represent the initial temperature of the fruit and the set point temperature of the delivery air in the associated cold chain unit operations, respectively. *T_fr_*(*t*) represents the fruit temperature, which can be, for example, the core temperature of the fruit pulp or the volume-averaged fruit temperature. Only the average temperature in this study is available and equal to the surface or core temperatures. The reason is that the two-phase model cannot capture possible thermal gradients inside the fruit. Given the relatively low airspeeds and Biot numbers, the thermal gradients will be limited, so the average fruit temperature is representative for the entire fruit.

From the definition of *Y*, the seven-eighths cooling/heating time (SECT, *t_7/8_*) and the half cooling time (HCT, *t_1/2_*) can be determined. The *t_7/8_* is the time required to reduce the temperature difference between the initial temperature of the fruit and that of the set point/delivery air by seven-eighths (*Y* = 0.125). The SECT is a useful parameter to characterize the cooling behavior of the fruit in each of the unit operations. The SECT is frequently used in commercial (pre)cooling operations because the fruit temperature at that value is acceptably close to the required storage temperature [71].

#### Shelf-life prediction

The remaining shelf life can be predicted based on the remaining quality at the end of the supply chain (A_i_(t_end_)) and the quality where the food is considered to be lost (A*_end,sl_* = 20 %). This remaining shelf life or keeping quality [79] is the remaining time until a commodity becomes unacceptable at shelf life conditions. The remaining shelf life can be predicted by taking the known final quality at the end of the supply chain, prior to shelf life storage, A(t_end_), and by keeping the fruit as of that point in time at constant conditions until A*_end,sl_* is reached. The shelf-life temperature used in this study is 23°C. At a constant shelf-life temperature, the rate constant k_i_ also remains constant, and the quality attribute shows an exponential decrease for the shelf-life prediction (for first-order reactions):

(64) $$A_{i}\left(t\right)=A_{0,i}e^{-k_{i}\left(T\right)t}+C_{i}$$

We reset the time for the shelf life calculation t' to zero at the start of the calculation, so t' = t-t_end_. Combining this equation with the known points (t'=0, A_i_= A_i_(t_end_)), (t' = t_RSL_, A_i_ = A*_end,sl_* = 20 %), C = 0 (assume no shift) and $k_{i}\left(T=T_{SL}\right)=k_{0}e^{\frac{-E_{a}}{RT_{SL}}}$, we get:

(65) $$A_{i}\left(t^{\prime }=0\right)=A_{0,i}=A_{i}\left(t_{end}\right)$$

(66) $$A_{i}\left(t_{RSL}\right)=A_{0,i}e^{-k_{i}\left(T_{SL}\right)t_{RSL}}=20\%$$

Out of these equations, we calculate t_RSL_ by:

(67) $$\begin{array}{c} A_{i}\left(t_{RSL}\right)=A_{i}\left(t_{end}\right)e^{-k\left(T_{SL}\right)t_{RSL}}=20\%\\ t_{RSL}=-\frac{1}{k(T_{SL})}\ln\left(\frac{A_{i}\left(t_{RSL}\right)}{A_{i}\left(t_{end}\right)}\right)=\frac{1}{k(T_{SL})}\ln\left(\frac{A_{i}\left(t_{end}\right)}{A_{i}\left(t_{RSL}\right)}\right)\\ t_{RSL}=-\frac{1}{k(T_{SL})}\left(\ln\left(A_{i}\left(t_{RSL}\right)\right)-\ln\left(A_{i}\left(t_{end}\right)\right)\right)=\frac{1}{k(T_{SL})}\left(\ln\left(\frac{1}{A_{i}\left(t_{RSL}\right)}\right)+\ln\left(A_{i}\left(t_{end}\right)\right)\right) \end{array}$$

### Comparison of porous medium simulations with experiments

#### Comparison with discrete CFD simulation of a pallet for validation of airflow and cooling

We compare the porous-media simulation for cooling a stack of palletized cartons with that of a fully-resolved CFD simulation [36]. This comparison allows accuracy verification of our porous media simulations. The simulations evaluate the cooling of orange fruit. The fruit are initially at ambient temperature (20°C) and cooled to -0.5°C, similar to the fully-resolved CFD simulations. The pressure of 1 Pa was imposed at the computational domain's inlet, leading to a superficial airspeed of 0.0235 m s^−1^, which is representative of airflow in a refrigerated container. The lateral boundaries were modeled as a symmetry boundary condition (slip wall), which assumes that the normal velocity component and the normal gradients at the boundary are zero. The average fruit temperatures for each of the 8 cartons are monitored for 100 h, so roughly 4 days. The fully-resolved CFD simulations did not include the thickness of the carton boxes, and only the fruit contributed to the porosity. Therefore, the porosity for airflow was adjusted, compared to the container simulations (ε = 38 %), and equals 47 % (= ε_fr_).

##### Full-scale experiment

We compare the cooling behavior of a simulated refrigerated container during ambient loading with a previous full-scale experiment we performed [75]. We aim to show that the simulations capture the cooling process sufficiently accurately. The experimental details are detailed in [75]. The experiment evaluates the cooling of fruit at ambient temperatures. The fruit was loaded in a refrigerated container at around 16°C and cooled there. Twenty pallets of 'Washington Navel' oranges fruit are loaded into Supervent packages. A void plug was installed at the bottom of the last pallets to reduce flow, bypassing the pallets. The delivery air temperature and return air temperature were monitored. The air set temperature was 1°C. However, as an input for the simulations, the measured delivery air temperature was imposed at the inlet. The fruit core temperatures in a carton are monitored in 6 pallets at 3 different heights. The shipment was monitored for about 21 days. The results of these validation experiments are discussed in the accompanying paper.

## Results

### Validation of the porous-medium model

**Fig. 20:**
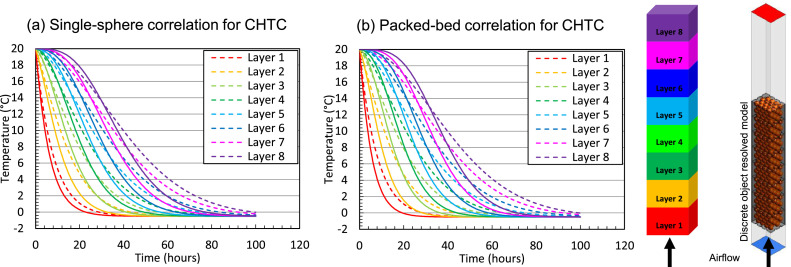
The volume-averaged temperature of all fruit in a specific carton for each layer of cartons as a function of time using a CHTC correlation for (a) a single sphere and (b) a packed bed. Dashed lines are data from the discrete object-resolved approach, and full lines are the porous-medium approach.

We verify the accuracy of the physics-based porous media simulations. Therefore, we compare the porous-media simulation for cooling a stack of palletized cartons with a fully-resolved CFD simulation of this geometry [36]. The average fruit pulp temperature of each of the eight layers of fruit in a carton is shown in Fig. 20 for both porous-medium and discrete object-resolved simulations. We included a comparison for simulations with a single-sphere correlation and a packed-bed correlation. The predicted airflow rate through the stack of cartons, for the imposed pressure difference over the stack, was below 1 % of the discrete object-resolved simulations.

When using the single-sphere correlation, a satisfactory agreement in cooling time and shape of the cooling curves is obtained. Still, the porous medium simulations cool down a bit faster than the CFD simulations. When using the packed bed correlation, we get a too-high cooling behavior using the porous medium approach, mainly of the bottom cartons of fruit. The reasons for the discrepancies between discrete and porous-medium simulations are the following:

• The same CHTC-U correlation is assumed for all fruit in the entire pallet for the porous-medium approach. In contrast, the local relation between the CHTC and the airspeed will differ from carton to carton in the discrete model.
• The porous-medium approach assumes a well-ventilated bed. In reality, fruit is packed. This packaging has little ventilation holes, by which several zones in the packaging are not well ventilated, and some of the fruit will cool less fast and also with different kinetics than in a homogeneous packed bed. This could explain why the porous medium model predicts higher heat transfer rates.
• The packed bed correlation we used was derived from an entire packed bed based on the approach-flow temperature. However, in our implemented porous-medium modeling approach, we use this correlation to calculate the local CHTC at each location in the packed bed based on the local airspeed. We also use the local air temperature to calculate the local heat flux from the fruit. As such, using the packed-bed correlation for the CHTC of the full packed bed is likely less representative of the local CHTC and temperature we are modeling. The reason is that this correlation relates the heat flow from the fruit to the approach flow air temperature and not to the local air temperature.

All these differences are responsible for the different cooling times and kinetics (shape of the cooling curves). In this study, we used the single-sphere correlation. This correlation accounts for the local temperature close to the product. Since this single-sphere correlation gives an acceptable agreement with the discrete object-resolved simulations, we can conclude that it is acceptable to use it as an approximation for closely packed spheres in ventilated packaging.

### Exploratory findings from simulations

This section gives an overview of our findings while setting up and troubleshooting the simulations. We report qualitative findings and do not provide additional data. This is because these simulations were exploratory, and we did not explicitly investigate each parameter in detail. However, the aim is to create awareness for modelers of specific hurdles we faced.

### Physics of airflow

• **2D vs 3D**. 3D simulations seem to have a roughly similar cooling behavior as our 2D simulations.
• **Laminar vs. turbulent flow**. Turbulent flow significantly increases the pressure losses over the cargo compared to laminar flow.
• **Coanda effect**. A larger inlet relaxes the Coanda effect for turbulent flow and makes the flow remains less attached to the bottom wall.
• **Inlet geometry.** The shape of the inlet did not significantly affect the cargo hold's flow field significantly.
• **Baffle plate.** The presence of a baffle plate did not significantly affect the overall airflow field. Locally, in the vicinity of the baffle plate, the airflow field was different.

### Simulation stability

• **T-bar floor**. Modeling the T-bar floor as a thin porous medium (screen or perforated plate) gave rise to instabilities in the simulation. The reason is likely that there is a significant change in airflow direction, namely from horizontal flow in the cavity below the T-bar floor to vertical into the pallets of fruit. It is challenging for perforated-plate models to capture this change in the flow direction. As a result, even screens with very small resistances led to convergence problems. Using a larger height of the T-bar floor improved numerical stability.
• **Grid**. It was more challenging to have proper convergence using a structured grid built out of quadrilateral elements than a triangular grid. However, quadrilateral boundary-layer elements were used on the walls as boundary-layer elements.
• **Porous-medium parameters**. To increase numerical stability, it helped to ramp up the magnitude of the porous medium parameters, for example, by starting from 10 % of the pressure resistance, in a few steps, to its full resistance.
• **Airspeed**. Ramping up the airspeed in several steps helped to avoid numerical stability issues.

The physics of the recirculation zone predicted in the pallets for turbulent flow [99].

• **T-bar and inlet height.** Increasing the height of the T-bar floor and inlet to about twice its normal height (63.5 mm) removed the recirculation zone while keeping the airflow rate through the slot. The slot Reynolds number, however, stays the same as the airspeed decreases with the same factor.
• **Slot Reynolds number.** Reducing the airspeed, so the slot Reynolds number, reduces the size of the recirculation zone.
• **Void plug**. The presence of the void plug did not impact the occurrence of a recirculation zone. Hence blocking the rear cavity to avoid airflow bypass near the door end has little impact.
• **Laminar flow**. Creeping airflow (Stokes flow) instead of turbulent airflow does not lead to a recirculation zone in the cargo [99]. No Coanda effect (so jet attachment) is observed.
• **T-bar floor**. Modeling the T-bar floor as a thin porous medium (screen or perforated plate) did not remove the recirculation zone and introduced challenges in convergence. Modeling the T-bar floor with discrete interior walls did not remove the recirculation zone but was more numerically stable. Others also modeled the T-bar floor discretely and found a recirculation zone [45,12]. Not modeling the T-bar floor reduced the size of the recirculation zone since the air jet is less restricted to remain inside the T-bar cavity and can move up more easily. The T-bar floor seems to enhance the presence of a recirculation zone. Modeling the T-bar floor as an isotropic porous medium of a few centimeters thick removed the recirculation zone. However, this representation is not entirely correct as the airflow resistance in the horizontal direction is rather small in reality.
• **The gap between the pallet and the wall at the refrigeration-unit end**. The presence of this gap did not affect the recirculation zone.
• **The turbulence intensity at the inlet** did not affect the recirculation zone.

## An outlook for future physics-based model development

The current physics-based model can be extended to increase insights and improve accuracy.

Turbulent airflow calculations are an essential future step to better capture the airflow, particularly in the channels upstream and downstream of the pallets. However, turbulent airflow was found in simulations to predict a recirculation zone in the first pallets at the refrigeration unit end. This could lead to a zone that cools down slower in these pallets [99]. The presence of this recirculation zone was not found in experiments explicitly yet. The current mismatch between simulated and experimental flow and thermal fields must be clarified first.

We also have a slight inconsistency when using the porous medium approach to calculate flow in the pallets of fruit is only valid under certain conditions [99]. The representative elementary volume (REV, computational cell) should be larger than the pore size or fruit diameter when using the porous medium approach. This is not the case for large fruit in pallets in refrigerated containers. The alternative is explicitly modeling the thousands of fruit and hundreds of ventilated packages with vent holes. This scenario is computationally very expensive. One of our previous works required 40 million cells [100] to simulate all orange fruit in a single pallet.

Various other physics-based model extensions include: (1) modeling the container in 3D, which is likely to affect the cooling at the sides of the pallets at the container walls predominantly; (2) exploring the impact of buoyancy on the airflow and heat transfer in the cargo, which is only relevant at the start of cooling, since then the temperature differences between the fruit surfaces and the air are the largest; (3) including the fan and other components, such as the evaporator heat exchanger, in the return air duct in the refrigerated container [12,101]; (4) composing a library of porous media properties of different fruit packed in ventilated packaging, since the packaging type and loading density will affect the pressure resistance of the pallets, and thereby also the airflow field and heat transfer, to some extent.

We can extend the fruit quality predictions to include more relevant quality attributes. Examples are chilling injury incidence, pest mortality, mass loss of the fruit, occurrence of condensation, and the associated risk of microbiological decay, and for other fruit firmness and total soluble solids and acidity, for example. Note that our models do not aim to achieve very high absolute accuracy for fruit quality modeling. The models are calibrated, but inherently we have variability in the models as the fruit properties vary with harvest year and location due to differences in breeding and growing conditions [25]. Another source of variability is that even fruit from the same orchard that are packed in the same container can exhibit differences in initial quality due to biological variability. The resulting induced inaccuracies lead to physics-based models not being readily adopted in commercial operations. Now, only a relative qualitative comparison of different shipments is possible. However, when stakeholders appreciate the value of this relative ranking, the results can still be very valuable for decision-making. The decision remains still at the discretion of the stakeholder, but they have more indicators to base their decision on, compared to, for example, a time-temperature curve only.

## Ethics statements

None required.

## CRediT authorship contribution statement

**Thijs Defraeye:** Conceptualization, Methodology, Validation, Formal analysis, Investigation, Writing – original draft, Visualization, Supervision, Project administration, Funding acquisition. **Celine Verreydt:** Writing – review & editing, Methodology, Investigation, Conceptualization. **Julien Gonthier:** Writing – review & editing, Methodology, Investigation, Validation, Conceptualization. **Leo Lukasse:** Writing – review & editing, Methodology, Conceptualization. **Paul Cronjé:** Writing – review & editing, Methodology, Conceptualization. **Tarl Berry:** Writing – review & editing, Methodology, Conceptualization.

## Declaration of competing interest

The authors declare that they have no known competing financial interests or personal relationships that could have appeared to influence the work reported in this paper.

## Data Availability

Data will be made available on request.

## References

[1] Defraeye T., Lambrecht R., Delele M.A., Tsige A.A., Opara U.L., Cronjé P., Verboven P., Nicolai B. (2014). Forced-convective cooling of citrus fruit: cooling conditions and energy consumption in relation to package design. J. Food Eng..

[2] Defraeye T., Lambrecht R., Tsige A.A., Delele M.A., Opara U.L., Cronjé P., Verboven P., Nicolai B. (2013). Forced-convective cooling of citrus fruit: package design. J. Food Eng..

[3] EPAL, 2021. EPAL 2 pallet [WWW Document]. URL https://www.epal-pallets.org/eu-en/load-carriers/epal-2-pallet.

[4] Lawton, R., 2016. How refrigerated containers work?

[5] Getahun S., Ambaw A., Delele M., Meyer C.J., Opara U.L. (2018). Experimental and numerical investigation of airflow inside refrigerated shipping containers. Food Bioprocess Technol..

[6] Wild Y., Scharnow R., Rühmann M. (2005).

[7] Hamburd-Sud, 2021. Stay cool - we care.

[8] CRT, 2013. Cambridge refrigeration technology - personal communication -Performance Data for Marine Integral Refrigeration Units.

[9] Lukasse L., Harkema H., Otma E., Paillart M. (2012).

[10] Lukasse L.J.S., Baerentz M.B., Kramer-Cuppen J.E.D. (2011). Proceedings of the 23rd IIR International Congress of Refrigeration.

[11] Berry, T.M., Tiamiyu, N.A., van Zyl, J., Opara, U.L., Cronje, P., Ambaw, A., Defraeye, T., Coetzee, C., 2023. Fruit cooling performance within a fully loaded refrigerated container: CFD modelling and validation.

[12] Tiamiyu N.A. (2020).

[13] Verboven P., Flick D., Nicolaï B.M., Alvarez G. (2006). Modelling transport phenomena in refrigerated food bulks, packages and stacks: basics and advances. Int. J. Refrig..

[14] ASHRAE (2010). ASHRAE Handbook: Refrigeration (SI.

[15] De Castro L.R., Vigneault C., Cortez L.A.B. (2005). Effect of container openings and airflow rate on energy required for forced-air cooling of horticultural produce. Can. Biosyst. Eng..

[16] Baird C.D., Gaffney J.J., Talbot M.T. (1988). Design criteria for efficient and cost effective forced air cooling systems for fruits and vegetables. ASHRAE Trans..

[17] Ferrua M.J., Singh R.P. (2011). Improved airflow method and packaging system for forced-air cooling of strawberries. Int. J. Refrig..

[18] ASHRAE, 2012. ASHRAE Handbook - HVAC Systems and Equipment.

[19] Ogawa, A., 2021. Psychrometric chart at sea level in SI units [WWW Document]. URL https://commons.wikimedia.org/wiki/File:PsychrometricChart-SeaLevel-SI.jpg.

[20] Fitzgerald W.B., Howitt O.J.A., Smith I.J., Hume A. (2011). Energy use of integral refrigerated containers in maritime transportation. Energy Policy.

[21] James C. (2019). Sustainable Food Supply Chains: Planning, Design, and Control Through Interdisciplinary Methodologies.

[22] Defraeye T., Verboven P., Opara U.L., Nicolai B., Cronjé P. (2015). Feasibility of ambient loading of citrus fruit into refrigerated containers for cooling during marine transport. Biosyst. Eng..

[23] CP, 2020. Captain Peter for Remote Container Management [WWW Document]. URL https://remotecontainermanagement.com/(accessed 3.9.20).

[24] Freitag M. (2021). Dynamics in logistics. Dyn. Logist..

[25] Jedermann R., Praeger U., Lang W. (2017). Challenges and opportunities in remote monitoring of perishable products. Food Packag. Shelf Life.

[26] Maersk, 2020. Remote container management [WWW Document]. URL https://www.maersk.com/solutions/shipping/remote-container-management (accessed 3.9.20).

[27] Follett P.A., Neven L.G. (2006). Current trends in quarantine entomology. Annu. Rev. Entomol..

[28] Hattingh V., Moore S., Kirkman W., Goddard M., Thackeray S., Peyper M., Sharp G., Cronjé P., Pringle K., Cha D.H. (2020). An improved systems approach as a phytosanitary measure for thaumatotibia leucotreta (Lepidoptera: tortricidae) in export citrus fruit from South Africa. J. Econ. Entomol..

[29] Manrakhan A., Daneel J.H., Stephen P.R., Hattingh V. (2022). Cold tolerance of immature stages of Ceratitis Capitata and Bactrocera Dorsalis (Diptera: Tephritidae). J. Econ. Entomol..

[30] Moore S.D., Peyper M., Kirkman W., Marsberg T., Albertyn S., Stephen P.R., Thackeray S.R., Grout T.G., Sharp G., Sutton G., Hattingh V. (2022). Efficacy of various low temperature and exposure time combinations for thaumatotibia leucotreta (Meyrick) (Lepidoptera: Tortricidae) Larvae. J. Econ. Entomol..

[31] Delele M.A., Ngcobo M.E.K., Getahun S.T., Chen L., Mellmann J., Opara U.L. (2013). Studying airflow and heat transfer characteristics of a horticultural produce packaging system using a 3-D CFD model. Part I: model development and validation. Postharvest Biol. Technol..

[32] Gruyters W., Van De Looverbosch T., Wang Z., Janssen S., Verboven P., Defraeye T., Nicolaï B.M. (2020). Revealing shape variability and cultivar effects on cooling of packaged fruit by combining CT-imaging with explicit CFD modelling. Postharvest Biol. Technol..

[33] Han J., Zhao C., Yang X., Qian J., Fan B. (2015). Computational modeling of air flow and heat transfer in a vented box during cooling: optimal package design. Appl. Therm. Eng..

[34] O'Sullivan J., Ferrua M.J., Love R., Verboven P., Nicolai B., East A. (2016). Modelling the forced-air cooling mechanisms and performance of polylined horticultural produce. Postharvest Biol. Technol..

[35] Berry T.M., Defraeye T., Ambaw A. (2022). Exploring novel carton footprints for improved refrigerated containers usage and a more efficient supply chain. Biosyst. Eng..

[36] Defraeye T., Cronjé P., Verboven P., Opara U.L., Nicolai B. (2015). Exploring ambient loading of citrus fruit into reefer containers for cooling during marine transport using computational fluid dynamics. Postharvest Biol. Technol..

[37] Wu W., Cronjé P., Nicolai B., Verboven P., Linus Opara U., Defraeye T. (2018). Virtual cold chain method to model the postharvest temperature history and quality evolution of fresh fruit – a case study for citrus fruit packed in a single carton. Comput. Electron. Agric..

[38] Wu W., Cronje P., Verboven P., Defraeye T. (2019). Unveiling how ventilated packaging design and cold chain scenarios affect the cooling kinetics and fruit quality for each single citrus fruit in an entire pallet. Food Packag. Shelf Life.

[39] Ambaw A., Verboven P., Delele M.A., Defraeye T., Tijskens E., Schenk A., Nicolai B.M. (2013). CFD modelling of the 3D spatial and temporal distribution of 1-methylcyclopropene in a fruit storage container. Food Bioprocess Technol..

[40] Delele M.A., Bessemans N., Gruyters W., Rogge S., Janssen S., Verlinden B.E., Smeets B., Ramon H., Verboven P., Nicolai B.M. (2019). Spatial distribution of gas concentrations and RQ in a controlled atmosphere storage container with pear fruit in very low oxygen conditions. Postharvest Biol. Technol..

[41] Wu W., Defraeye T. (2018). Identifying heterogeneities in cooling and quality evolution for a pallet of packed fresh fruit by using virtual cold chains. Appl. Therm. Eng..

[42] Ambaw A., Verboven P., Delele M.A., Defraeye T., Tijskens E., Schenk A., Verlinden B.E., Opara U.L., Nicolai B.M. (2014). CFD-Based analysis of 1-MCP distribution in commercial cool store rooms: porous medium model application. Food Bioprocess Technol..

[43] Hoang H.M., Duret S., Flick D., Laguerre O. (2015). Preliminary study of airflow and heat transfer in a cold room filled with apple pallets: comparison between two modelling approaches and experimental results. Appl. Therm. Eng..

[44] Defraeye T. (2014). Advanced computational modelling for drying processes - a review. Appl. Energy.

[45] Getahun S., Ambaw A., Delele M., Meyer C.J., Opara U.L. (2017). Analysis of airflow and heat transfer inside fruit packed refrigerated shipping container: part I – Model development and validation. J. Food Eng..

[46] ASHRAE, 2010. ASHRAE handbook - refrigeration: systems and applications (SI edition). Atlanta.

[47] Helmig R., Flemisch B., Wolff M., Ebigbo A., Class H. (2013). Model coupling for multiphase flow in porous media. Adv. Water Resour..

[48] Ho Q.T., Carmeliet J., Datta A.K., Defraeye T., Delele M.A., Herremans E., Opara L., Ramon H., Tijskens E., Van Der Sman R., Van Liedekerke P., Verboven P., Nicolaï B.M. (2013). Multiscale modeling in food engineering. J. Food Eng..

[49] Ambaw A., Verboven P., Defraeye T., Tijskens E., Schenk A., Opara U.L., Nicolai B.M. (2013). Porous medium modeling and parameter sensitivity analysis of 1-MCP distribution in boxes with apple fruit. J. Food Eng..

[50] Berry T.M., Ambaw A., Defraeye T., Coetzee C., Opara U.L. (2019). Moisture adsorption in palletised corrugated fibreboard cartons under shipping conditions: a CFD modelling approach. Food Bioprod. Process..

[51] Ambaw A., Verboven P., Defraeye T., Tijskens E., Schenk A., Opara U.L., Nicolai B.M. (2013). Porous medium modeling and parameter sensitivity analysis of 1-MCP distribution in boxes with apple fruit. J. Food Eng..

[52] Datta A.K. (2007). Porous media approaches to studying simultaneous heat and mass transfer in food processes. I: problem formulations. J. Food Eng..

[53] Datta A.K. (2007). Porous media approaches to studying simultaneous heat and mass transfer in food processes. II: property data and representative results. J. Food Eng..

[54] Naghash M., Fathieh F., Besant R.W., Evitts R.W., Simonson C.J. (2016). Measurement of convective heat transfer coefficients in a randomly packed bed of silica gel particles using IHTP analysis. Appl. Therm. Eng..

[55] Wakao N., Kaguei S., Funazkri T. (1979). Effect of Fluid dispersion coefficients on particle-to-fluid heat transfer coefficients in packed beds. Chem. Eng. Sci..

[56] Alvarez G., Bournet P.E., Flick D. (2003). Two-dimensional simulation of turbulent flow and transfer through stacked spheres. Int. J. Heat Mass Transf..

[57] Ergun S. (1952). Fluid flow through packed columns. J. Chem. Eng. Prog..

[58] Getahun, S., 2017. Investigating cooling performance and energy utilization of refrigerated shipping container packed with fresh fruit using computational fluid dynamics modelling 218.

[59] Getahun S., Ambaw A., Delele M., Meyer C.J., Opara U.L. (2017). Analysis of airflow and heat transfer inside fruit packed refrigerated shipping container: part II – Evaluation of apple packaging design and vertical flow resistance. J. Food Eng..

[60] Dean R.B. (1978). Reynolds number dependence of skin friction and other bulk flow variables in two-dimensional rectangular duct flow. J. Fluids Eng..

[61] Senguttuvan S., Youn J.S., Park J., Lee J., Kim S.M. (2020). Enhanced airflow in a refrigerated container by improving the refrigeration unit design. Int. J. Refrig..

[62] BuffersUSA, 2022. Container Hardware Catalog 2022 [WWW Document]. URL https://www.buffersusa.com/FlipBuilder/container/mobile/index.html.

[63] Moureh J., Tapsoba M., Flick D. (2009). Airflow in a slot-ventilated enclosure partially filled with porous boxes: part II - measurements and simulations within porous boxes. Comput. Fluids.

[64] Defraeye T., Wu W., Prawiranto K., Fortunato G., Kemp S., Hartmann S., Cronje P., Verboven P., Nicolai B. (2017). Artificial fruit for monitoring the thermal history of horticultural produce in the cold chain. J. Food Eng..

[65] Casey, M., Wintergerste, T., 2000. Special interst group on “quality and trust in industrial CFD” Best Practice Guidelines, First edit. ed. ERCOFTAC.

[66] Kang L., van Hooff T. (2022). Influence of inlet boundary conditions on 3D steady RANS simulations of non-isothermal mechanical ventilation in a generic closure. Int. J. Therm. Sci..

[67] Defraeye T., Blocken B., Carmeliet J. (2010). CFD analysis of convective heat transfer at the surfaces of a cube immersed in a turbulent boundary layer. Int. J. Heat Mass Transf..

[68] Defraeye T., Verboven P., Nicolai B. (2013). CFD modelling of flow and scalar exchange of spherical food products: turbulence and boundary-layer modelling. J. Food Eng..

[69] Defraeye T., Blocken B., Derome D., Nicolai B., Carmeliet J. (2012). Convective heat and mass transfer modelling at air-porous material interfaces: overview of existing methods and relevance. Chem. Eng. Sci..

[70] Kondjoyan A. (2006). A review on surface heat and mass transfer coefficients during air chilling and storage of food products. Int. J. Refrig..

[71] Brosnan T., Sun D.W. (2001). Precooling techniques and applications for horticultural products - a review. Int. J. Refrig..

[72] Sadashive Gowda B., Narasimham G.S.V.L., Krishna Murthy M.V. (1997). Forced-air precooling of spherical foods in bulk: a parametric study. Int. J. Heat Fluid Flow.

[73] Defraeye T., Tagliavini G., Wu W., Prawiranto K., Schudel S., Assefa Kerisima M., Verboven P., Bühlmann A. (2019). Digital twins probe into food cooling and biochemical quality changes for reducing losses in refrigerated supply chains. Resour. Conserv. Recycl..

[74] Kader, A., 1997. Mango - Recommendations for maintaining postharvest quality [WWW Document]. UC Davis Commod. fact sheets. URL http://postharvest.ucdavis.edu/Commodity_Resources/Fact_Sheets/Datastores/Fruit_English/?uid=37&ds=798 (accessed 6.27.18).

[75] Defraeye T., Nicolai B., Kirkman W., Moore S., Niekerk S.V.S., Verboven P., Cronjé P. (2016). Integral performance evaluation of the fresh-produce cold chain: a case study for ambient loading of citrus in refrigerated containers. Postharvest Biol. Technol..

[76] Shrivastava C., Berry T., Cronje P., Schudel S., Defraeye T. (2022). Digital twins enable the quantification of the trade-offs in maintaining citrus quality and marketability in the refrigerated supply chain. Nat. Food.

[77] Ladaniya M.S. (2008).

[78] Owoyemi A., Porat R., Lichter A., Doron-faigenboim A., Jovani O., Koenigstein N., Salzer Y. (2022). Evaluation of the storage performance of ‘valencia’ oranges and generation of shelf-life prediction models. Horticulturae.

[79] Tijskens L.M.M., Polderdijk J.J. (1996). A generic model for keeping quality of vegetable produce during storage and distribution. Agric. Syst..

[80] Hertog M.L.A.T.M., Uysal I., Verlinden B.M., Nicolaï B.M. (2014). Shelf life modelling for first-expired-first-out warehouse management. Philos. Trans. R. Soc. A.

[81] Robertson G.L. (2016).

[82] Van Boekel M.A.J.S. (2008). Kinetic modeling of food quality: a critical review. Compr. Rev. Food Sci. Food Saf..

[83] Cantwell, M., 2001. Properties and recommended conditions for long-term storage of fresh fruits and vegetables.

[84] Lado J., Cronje P.J., Rodrigo M.J., Zacarías L. (2019). Postharvest Physiological Disorders in Fruits and Vegetables.

[85] Thompson J.F. (2004). USDA Agriculture Handbook Number 66: The Commercial Storage of Fruits, Vegetables, and Florist and Nursery Stocks.

[86] Defraeye T., Verboven P., Nicolai B. (2013). CFD modelling of flow and scalar exchange of spherical food products: turbulence and boundary-layer modelling. J. Food Eng..

[87] Tagliavini G., Defraeye T., Carmeliet J. (2019). Multiphysics modeling of convective cooling of non-spherical fruit to unveil quality evolution throughout the cold chain. J. Food Eng..

[88] Whitaker S. (1972). Forced convection heat transfer correlations for flow in pipes, past flat plates, single cylinders, single spheres, and for flow in packed beds and tube bundles. AIChE J..

[89] Imai T., Murayama T., Ono Y. (1994). The effect of structure of packed beds on the convective heat between particle and liquid. Iron Steel Inst. Jpn..

[90] Bon J., Váquiro H., Benedito J., Telis-Romero J. (2010). Thermophysical properties of mango pulp (Mangifera indica L. cv. Tommy Atkins). J. Food Eng..

[91] COMSOL, 2022. COMSOL Multiphysics software v6.0 - Reference manual - Material Library.

[92] Gruyters W., Defraeye T., Verboven P., Berry T., Ambaw A., Opara U.L., Nicolai B. (2019). Reusable boxes for a beneficial apple cold chain: a precooling analysis. Int. J. Refrig..

[93] Lufu R., Ambaw A., Berry T.M., Opara U.L. (2020). Evaluation of the airflow characteristics, cooling kinetics and quality keeping performances of various internal plastic liners in pomegranate fruit packaging. Food Packag. Shelf Life.

[94] CGA, 2022. Citrus Grower Association: Industry statistics - 2021 Export Season.

[95] Berry T.M., Defraeye T., Wu W., Sibiya M.G., North J., Cronje P.J.R. (2021). Cooling of ambient-loaded citrus in refrigerated containers: what impacts do packaging and loading temperature have?. Biosyst. Eng..

[96] Defraeye T., Nicolai B., Kirkman W., Moore S., Niekerk S.V., Verboven P., Cronjé P. (2016). Integral performance evaluation of the fresh-produce cold chain: a case study for ambient loading of citrus in refrigerated containers. Postharvest Biol. Technol..

[97] Defraeye T., Cronjé P., Berry T., Opara U.L., East A., Hertog M., Verboven P., Nicolai B. (2015). Towards integrated performance evaluation of future packaging for fresh produce in the cold chain. Trends Food Sci. Technol..

[98] Thompson (2008).

[99] Defraeye, T., Lukasse, L., Shrivastava, C., Verreydt, C., Schemminger, J., Cronje, P., Berry, T., 2022. Is there a systematic hidden “hot spot” in refrigerated containers filled with fresh food in ventilated packaging?

[100] Wu W., Cronjé P., Verboven P., Defraeye T. (2019). Unveiling how ventilated packaging design and cold chain scenarios affect the cooling kinetics and fruit quality for each single citrus fruit in an entire pallet. Food Packag. Shelf Life.

[101] Senguttuvan S., Rhee Y., Lee J., Kim J., Kim S. (2021). Enhanced heat transfer in a refrigerated container using an airflow optimized refrigeration unit. Int. J. Refrig..

